# Formation and Identification of Six Amino Acid - Acrylamide Adducts and Their Cytotoxicity Toward Gastrointestinal Cell Lines

**DOI:** 10.3389/fnut.2022.902040

**Published:** 2022-05-20

**Authors:** Dan Li, Fangfang Xian, Juanying Ou, Kaiyu Jiang, Jie Zheng, Shiyi Ou, Fu Liu, Qinchun Rao, Caihuan Huang

**Affiliations:** ^1^Department of Food Science and Engineering, Jinan University, Guangzhou, China; ^2^Institute of Food Safety & Nutrition, Jinan University, Guangzhou, China; ^3^Guangdong-Hong Kong Joint Innovation Platform for the Safety of Bakery Products, Guangzhou, China; ^4^Department of Nutrition and Integrative Physiology, Florida State University, Tallahassee, FL, United States

**Keywords:** acrylamide, amino acid, adduct, elimination mechanism, cytotoxicity

## Abstract

Acrylamide (AA) is a food contaminant, and amino acids are suggested to mitigate its toxicity by forming adducts. The emergence of acrylamide adducts may cause underestimation of acrylamide exposure level as well as trigger new safety problems. Based on the acrylamide elimination capability of four amino acids, this study chemically synthesized six amino acid-acrylamide adducts. Their structures were analyzed, followed by content determination in 10 commercially baking foods. The Michael adduct formed by one molecule of γ-aminobutyric acid (GABA) and acrylamide was most abundant in foods among six adducts. Furthermore, it markedly decreased the cytotoxicity of acrylamide in Caco-2 cells and GES-1 cells. This finding suggests that amino acids can be used to reduce acrylamide level in processed foods and mitigate its hazardous effects after intake.

## Introduction

Acrylamide (AA) is a contaminant present widely in thermally processed foods. It has been classified as a probable human carcinogen by the International Agency for Research on Cancer (IARC) (1) and raises health concerns with detrimental effects. As an undesirable product of Maillard reaction, it is produced at high temperatures from amino acid asparagine and reducing sugars, such as glucose and fructose (2). Therefore, high content of acrylamide is often detected in carbohydrate-rich foods, such as potato chips (12–3,241 μg/kg), breakfast cereals (167–726 μg/kg), and coffee (7.5–1190.2 μg/kg) (3, 4). According to the European regulation (Commission Regulation (EU) 2017/2,158), the benchmark of acrylamide in the above foods is 750, 300, and 850 μg/kg, respectively, which are far less than the actual levels of acrylamide. In this case, long term intake of foods with high acrylamide content will increase the risk of prostate cancer, breast cancer and ovarian cancer (5).

After ingestion, acrylamide can be metabolized to glycidamide, which, like acrylamide, can covalently bind to the N-terminal valine of hemoglobin (Hb) and form adducts. Hb adduct levels are associated with increased breast cancer risk, adverse peripheral nervous system symptoms, reduced serum insulin, etc (6, 7). Besides, acrylamide and glycidamide can also react with the –NH_2_ and –NH groups of purine and pyrimidine bases in DNA, inducing DNA damage and mutagenic consequences (7). In addition, acrylamide has been reported to present neurotoxic effects in the human workplace environment and reproductive toxicity as well as immunotoxicity in laboratory animals (8).

Considering the health risks associated with acrylamide ingestion, reducing acrylamide content in foods has become one of the most important issues in food safety. A number of strategies have been proposed to control acrylamide levels in processed foods, including replacing food materials with those has low acrylamide precursor content, such as selecting potato cultivars with low concentrations of reducing sugars or changing harvest time and storage conditions (9, 10); reducing the amount of acrylamide precursor, such as application of *L-*asparaginase in foods (11); pretreatment of food materials, such as washing olives with water at 25°C before lye and thermal treatment, or immersing potato slices in different solutions before frying (12, 13); regulation of food processing conditions, such as lower temperature and shorter processing time (14); and applying functional food ingredients to prevent acrylamide formation, such as amino acids. Kobayashi et al. (15) found lysine and cysteine mediated acrylamide content reduction in aqueous solution below 120°C. Koutsidis and his colleagues (16) reported that proline, tryptophan, glycine, and cysteine suppressed acrylamide formation by 45–80% after heating at 160°C for 60 min in the asparagine-glucose model system. Our previous studies found that cysteine, glycine and lysine could directly eliminate 69.6–94.6% of acrylamide in 15 min in the amino acid-acrylamide systems at 160°C (17). In the food system, soaking potato slices with 0.5% glycine decreased more than 70% acrylamide content in fried potato chips (18). These researches indicate that amino acids are ideal acrylamide eliminators and may be potent agents to reduce the negative effects of acrylamide.

The high elimination rate of amino acids was related to the formation of the amino acid-acrylamide adduct (16, 17, 19). The structures of adducts, however, have not been confirmed yet. Also, there is a need to find out the formation mechanism of acrylamide adducts, their presence in real food systems, and whether they are hazardous in foods. The present work aims to answer the above questions by synthesizing amino acid-acrylamide adducts and building relevant determination methods. Four amino acids were selected to react with acrylamide. γ-Aminobutyric acid (GABA) is a non-proteinogenic amino acid with high content in potatoes (6,460 μmol/kg), which plays an important role in preventing sleeplessness, promoting neuron growth, and controlling anxiety and depression (20). Glycine is the lowest molecular weight amino acid, required for various metabolic pathways, including glutathione synthesis and one-carbon metabolism (21). Lysine and tryptophan are essential amino acids in human nutrition involved in several physiological processes, including growth performance and regulation of the immune systems (22, 23). Their elimination capability to acrylamide, the structure and level of main products in foods, and cytotoxicity were investigated in the current study.

## Materials and Methods

### Materials

Acrylamide (AA, ≥99%), lysine (Lys, ≥98%), tryptophan (Trp, ≥99%), GABA (≥98%), glycine (Gly, ≥99.5%) and n-hexane were purchased from J&K Scientific Co., Ltd (Beijing, China). Methanol (HPLC), D_2_O and DMSO were purchased from Energy Chemical Reagent Co., Ltd (Shanghai, China). A-HG type octadecyl bonded 1 silica gel was purchased from YMC Co., Ltd (Tokyo, Japan). Dextran gel Sephadex LH-20 was purchased from Extrasynthese Co., Ltd (Lyon, France). 3-(4,5-Dimethylthiazol-2-yl)-2,5-diphenyltetrazolium bromide (MTT), RPMI-1640 medium, fetal bovine serum (FBS) and penicillin-streptomycin were purchased from Shanghai Zhongqiao Xinzhou Biotechnology Co., Ltd, (China). Ten food products (Potato chips A, Potato chips B, Potato chips C, brown rice cake, egg biscuit, cereal bars, dough twist, walnut cake and rice crust) were purchased from Utilize modern supermarkets (Guangzhou, China).

### Scavenging Capacity of Amino Acids for Acrylamide

The scavenging ability of four amino acids for acrylamide was studied in a model system. Four milliliters of reaction solution containing 50 mM amino acids (Lys, Trp, GABA, Gly) and 5 mM AA aqueous solutions were placed in a 10 mL stopper colorimeter and heated in a water bath at 80°C for 5 h. The samples were then cooled in an ice bath and analyzed with high performance liquid chromatography-diode array detector (HPLC-DAD) systems (UltiMate 3000, Thermo Fisher, Germany). The residual content of AA was determined using our previously reported method (13). Briefly, 5 μL of the filtered sample was injected into an Atlantis T3 column (4.6 mm × 250 mm, 5 μm, Waters Corporation, Milford, US) and isocratically eluted by methanol/water (2:98, v/v) solution at a flow rate of 0.4 mL min^−1^ and 40.0°C. AA residue was measured at 205 nm and quantified using an external standard curve.

The reaction products were further identified through HPLC-MS/MS analysis (LCMS-8045, Shimadzu Corporation, Kyoto, Japan) based on Hu et al. (21). The injection volume was 10 μL. HPLC procedure was applied as described above. MS/MS spectrum was acquired in positive ion mode with mass spectra over a range of m/z 50–800, source temperature of 300°C, desolvation temperature of 250°C and capillary voltage of 4.0 kV. The collision energy was set at 20.0 eV for product ion scans.

### Optimization of Reaction Conditions Between Acrylamide and Amino Acids

Forty milliliters of reaction solutions containing 50 mM amino acids and different levels of AA (6.25, 12.5, 25, 50 mM) were, respectively reacted in a 50 mL sealed conical flask at 95°C for 5 h. The samples were then cooled down in an ice bath and filtered through a 0.45 μm membrane (Jinteng Equiment Co. Ltd, Tianjin, China) for HPLC analysis as described above. The injection volume was 10 μL. Acrylamide and the adducts were detected at 205 nm and confirmed with LC-MS/MS as described above.

### Synthesis and Purification of Amino Acid-Acrylamide Adducts

Amino acids-acrylamide adducts were synthesized by mixing amino acids and acrylamide according to the method of Zhao et al. (24) with little modifications and the optimized reaction conditions were obtained from 2.3. Briefly, 100 mL of the aqueous solution containing a different ratio of acrylamide and amino acids were incubated at 80°C under constant stirring for 300 min. The ratio of amino acids to acrylamide (50 mM) was set as follows: Lys:AA = 1:1, Gly:AA = 1:2, GABA/Trp:AA = 1:4. At the end of the reaction, the products were concentrated with a vacuum rotary evaporator (Eyela N-1300, Tokyo, Japan), and the adducts in the concentrate were purified with an ODS C18 reverse silica gel column (Gly-AA and Lys-AA) or Sephadex LH-20 (Trp-AA and GABA-AA) monitored by HPLC (UltiMate 3000, Thermo Fisher, Germany). Elution solutions for different adducts were listed in Table 1, and the fractions containing more than 80% of the target adducts were collected and further purified with semi-preparative liquid chromatography (25). Separation was performed with a Pntulips QC-C18 column (250 × 10 mm, 5 μm, Puning Analytical Technology Co. Ltd, Shanghai, China) using a five-peak series liquid chromatography system (Wufeng Scientific Instrument Co., Ltd, Shanghai, China) consisting of a reciprocating double-plunger parallel pump and AN LC 100 ultraviolet detector. The elution solutions were presented in Table 1, and the purified adducts were evaporated using a rotary vacuum evaporator at 55°C, followed by freeze-drying with a scientZ-10N vacuum freeze-dryer (Xinzhi Biotechnology Co., Ltd, Ningbo, China).

**Table 1:** Condition of column chromatography and semi-preparative LC.

**Reaction**	**Column chromatography**		**Semi-preparative LC elution solution**
	**Column packing**	**Elution solution**	
		**(methanol:water)**	
Lys-AA	ODS	2: 98	methanol: 0.1% ammonia water = 9:91
Trp-AA	Sephadex LH-20	15:85	methanol: water = 2:98
GABA-AA	Sephadex LH-20	2: 98	methanol: water = 2:98
Gly-AA	ODS	2: 98	methanol: water = 2:98

### Structural Analysis of Purified Amino Acid-Acrylamide Adducts

The structures of purified adducts were identified with mass spectrometry (MS) analysis and nuclear magnetic resonance (NMR). An X500R QTOF high-resolution mass spectrometer (HRMS; AB Sciex, MA, United States) was operated at 500°C, with a capillary voltage of 5.5 kV, drying gas and nebulizer gas pressure of 50 and 55 psi, respectively. The sample was separated on an Atlantis T3 C18 column (4.6 × 150 mm, 5 μm, Waters Corporation, Milford, US). The spectrum was scanned in positive ion mode and obtained over a mass range from 50 to 1,000 Da.

For NMR analysis, 10 mg of the purified adducts were dissolved in 0.55 mL of D_2_O (Lys-AA, GABA-AA 1, Gly-AA 1 and Gly-AA 2) or DMSO-d_6_ (Trp-AA and GABA-AA 2) (20). ^1^H, ^13^C, Dept 135 and two-dimensional NMR spectra were obtained using a Bruker 600 MHz Avance III NMR spectrometer.

### Detection of the Adduct in Ten Commercially Available Baking Foods

Fifteen grams of crushed samples were defatted with n-hexane using vortex at 25°C. Four grams of defatted samples were ultrasonically extracted three times in a 50 mL centrifuge tube for 10 min with 10 mL of 30% methanol solution at 25°C and frequencies of 100 kHz, followed by centrifugation at 4,481 × g for 10 min at 25°C. The supernatants were combined and filtered through a 0.45 μm membrane for HPLC-MS/MS analysis (LC-MS 8045, Shimadzu Corp., Kyoto, Japan). Ten microliters of the sample were injected into the LC-MS system and separated on an Atlantis T3 C18 column with a flow rate of 0.4 mL/min at 40 °C. Elution was carried out with methanol (A) and water (B), and gradient flow was as followed: A = 2% at 0 ~ 6 min; A = 50% at 6.01 ~ 16 min; A = 2% at 16.01 ~ 23 min. A multiple reaction monitoring (MRM) method was applied to detect the adducts with mass spectrometry, which was operated in positive ion mode with the source temperature of 300°C, the de-solvation temperature of 250°C, the capillary voltage of 4,000 V, and the scanning rate of 1,000 Da/sec. The parent ions and daughter ions of each adduct were obtained by the first-stage full scan and the second-stage fragment ion analysis of mass spectrometry, followed by optimizing the collision voltage. Qualitative ion pair, quantitative ion pair and optimized voltage are shown in Table 2.

**Table 2:** Qualitative ion pair, quantitative ion pair and optimized voltage.

**Compounds**	**Ion pairs**	**Q1 voltage (V)**	**Collision voltage (V)**	**Q3 voltage (V)**
AA	72/55^*^ 72/27 72/44	−14 −12 −13	−15 −26 −25	−22 −29 −19
Gly-AA 1	147/88^*^ 147/101 147/60	−10 −15 −10	−14 −13 −18	−16 −18 −24
Gly-AA 2	218/159^*^ 218/88 218/130	−24 −11 −24	−13 −22 −18	−29 −16 −22
Lys-AA	218/142^*^ 218/114 218/84	−11 −11 −11	−16 −24 −22	−14 −21 −15
Trp-AA	276/188^*^ 276/144 276/230	−30 −29 −30	−16 −26 −13	−20 −14 -25
GABA-AA 1	175/98^*^ 175/116 175/140	−19 −21 −19	−19 −15 −13	−18 −20 −14
GABA-AA 2	246/187^*^ 246/84 246/101	−12 −12 −29	−15 −27 −22	−19 −15 −10

* *: Quantitative ion*.

### Cell Viability Assay

Human colon cancer epithelial cell line Caco-2 and Human gastric mucosal epithelial cell line GES-1 cell lines were applied to investigate the cytotoxicity of the adduct GABA-AA 1. Cells were cultivated with RPMI-1640 medium containing 10% FBS and 1% penicillin-streptomycin in an incubator with 5% CO_2_ at 37 °C. The MTT assay was performed for cell viability analysis. Briefly, cells were seeded in a 96-well-plate at a density of 5 × 10^3^/ well-overnight to allow sufficient attachment, and then treated with AA or GABA-AA 1 at 0.5, 1.0, 1.5, 2.0, 2.5, 3.0, 3.5, 4.0, 4.5 and 5.0 mM for 24 h. Cell viability was determined by the MTT assay described by Zou et al. (26).

### Statistical Analysis

All experiments were repeated at least three times. The data were presented as mean ± standard deviation (SD) and were analyzed with one-way ANOVA using SPSS statistical software (version 25.0, SPSS. Inc., Chicago, IL, USA). *P* < 0.05 was considered to be statistically significant.

## Results and Discussion

### Amino Acid Scavenges Acrylamide via Adduct Formation

**Figure 1:**
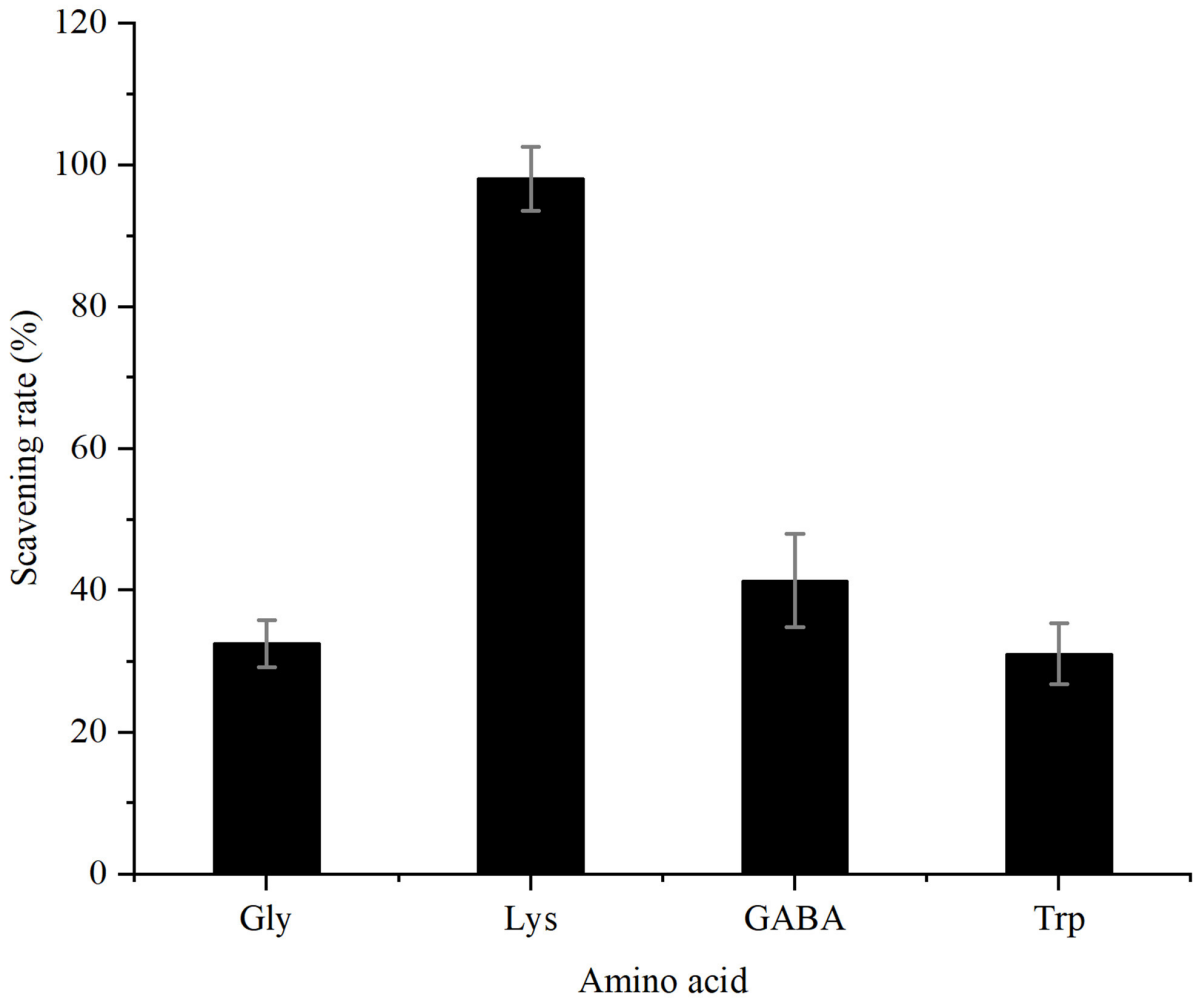
Scavenging capacity of four amino acids for acrylamide.

Model reactions containing acrylamide and different amino acids were performed at 80 °C for 5 h. The remaining acrylamide content was quantified using calibration curves obtained from HPLC-DAD and the scavenging rates were calculated as shown in Figure 1. Among the four amino acids, lysine had the highest elimination rate of acrylamide (98%), which was much higher than our previous results of 69.6% (17), since the reaction time was more sufficient. The other three amino acids showed lower elimination capability that 41, 32, and 31% of acrylamide was reduced by γ-aminobutyric acid, glycine and tryptophan, respectively.

**Figure 2:**
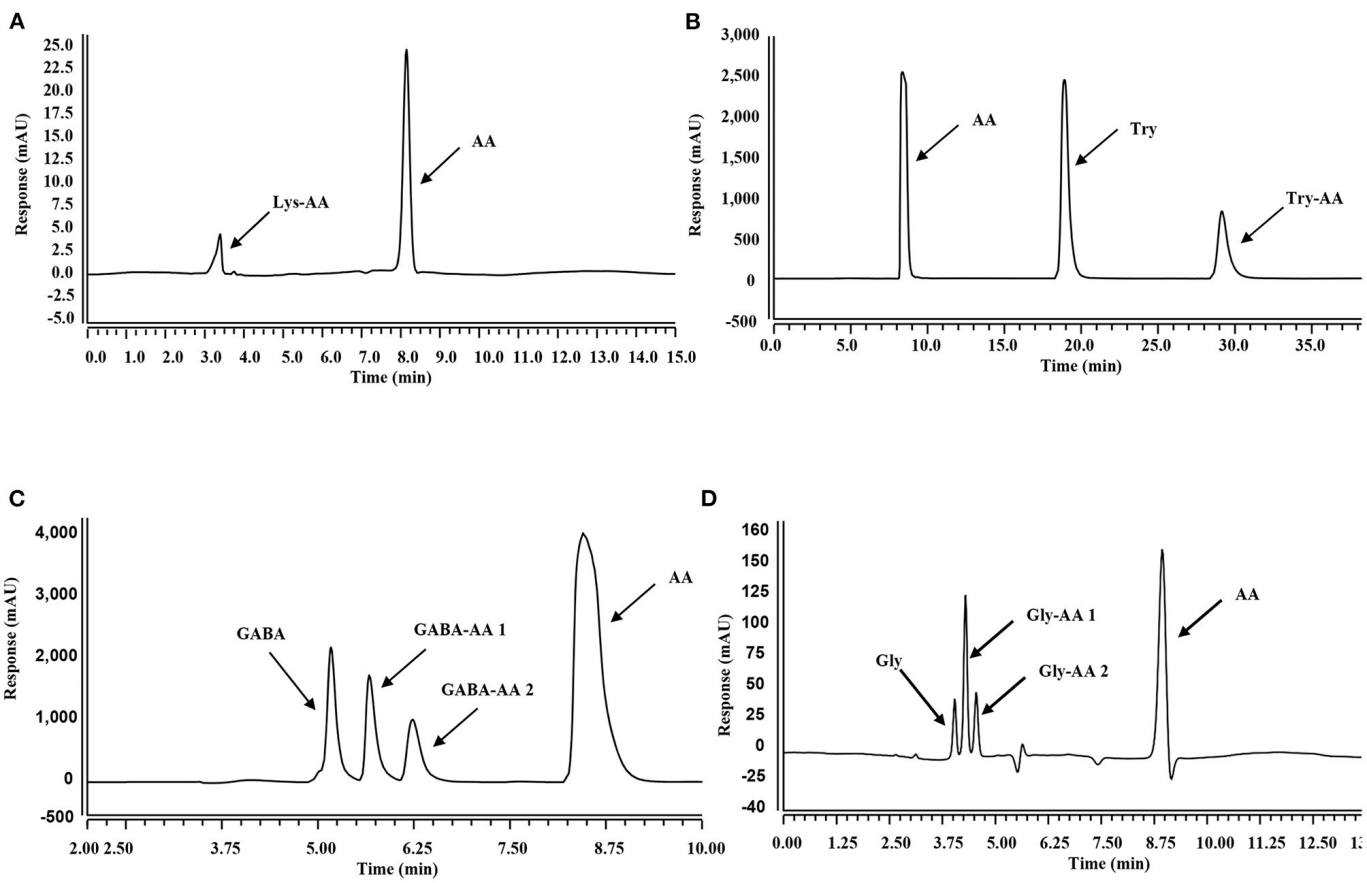
HPLC chromatographs of mixtures in different systems. **(A–D)**: Mixtures obtained from acrylamide and Lys **(A)**, Trp **(B)**, GaBa **(C)**, and Gly **(D)** co-incubation systems at 80°C for 300 min.

As displayed by the HPLC chromatograms in Figure 2, the decreased intensity of the acrylamide peak was accompanied by the emergence of new peaks, indicating the formation of new compounds. Zamora et al. (27) reported that acrylamide could react with nucleophilic amino groups on amino acid side chains to produce the corresponding Michael adducts. Therefore, these new substances were further analyzed with HPLC-MS/MS and were confirmed as acrylamide adducts (Supplementary Figures S1, S2).

### Optimization of Reaction Conditions for Amino Acid-Acrylamide Adducts Synthesis

**Figure 3:**
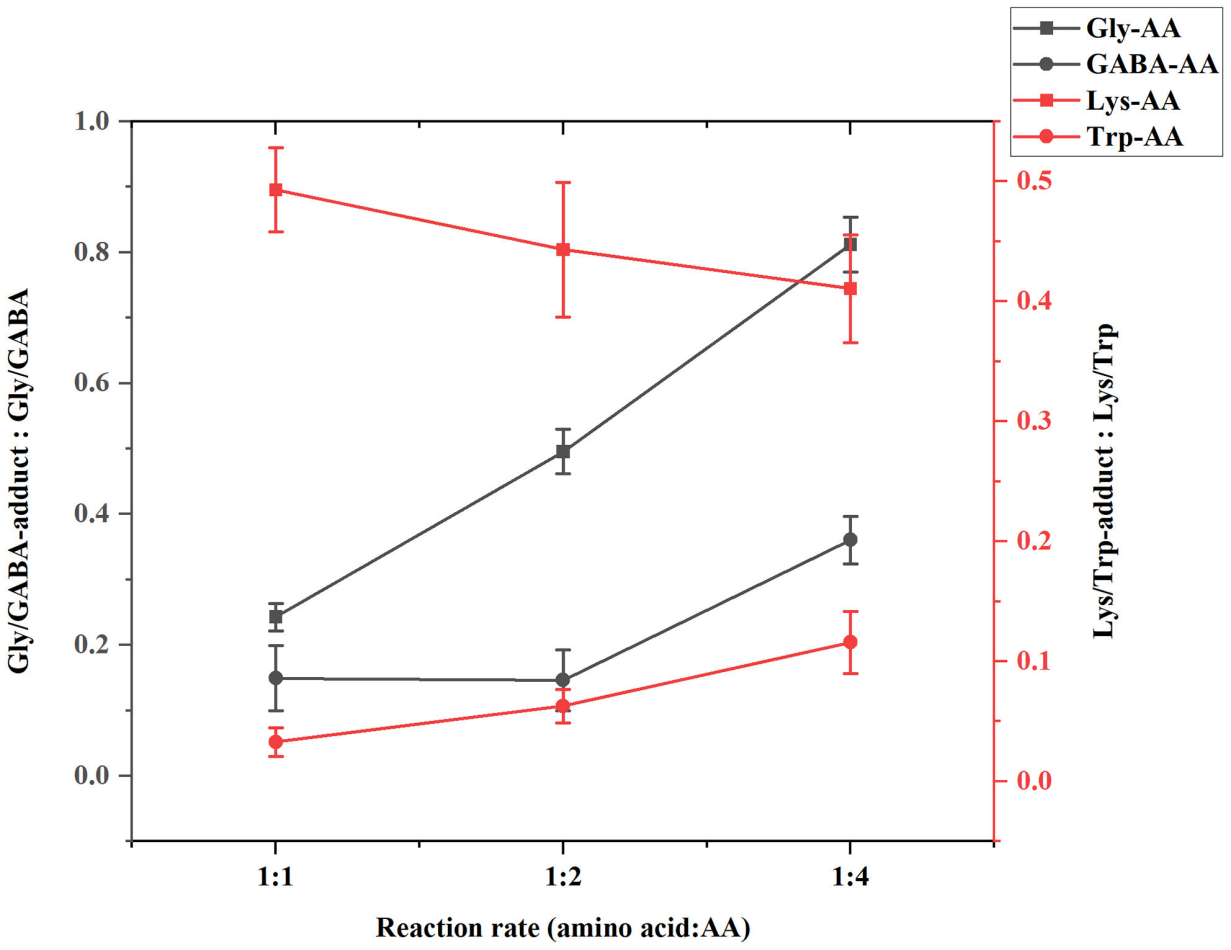
Target adduct to amino acid ratio under different amino acid and AA reaction ratios.

To obtain abundant adducts for further investigation, reaction conditions were studied for adducts synthesis. Three reaction ratios of amino acids to acrylamide (1:1, 1:2, 1:4) were performed to obtain more adducts and fewer remaining amino acids for isolation and purification. The optimum reaction ratio was determined by the peak area ratio of the adduct and remaining amino acids in HPLC chromatograms. As shown in Figure 3, with the increase of acrylamide concentration, the content of lysine adducts decreased, so the ratio of 1:1 was selected for lysine adduct synthesis. For GABA and tryptophan, 1:4 was chosen for reaction since the higher acrylamide concentration, the higher the adducts concentrations. In the glycine reaction system, although the adduct levels were in proportion to the remaining amino acids concentration, excessive acrylamide formed gel with glycine in the reaction solution. Therefore, the reaction ratio was 1:2 for glycine and acrylamide.

### Structural Analysis of the Amino Acid-Acrylamide Adducts

Six purified adducts were obtained since GABA and glycine formed two adducts with acrylamide. HRMS and NMR were subsequently performed to confirm the structures of these adducts. The wavelength, molecular weight and chemical formula of the adducts in the four reaction systems was shown in Table 3.

**Table 3:** Wavelength, m/z, and molecular formula of adducts.

**Adducts**	**Wavelength (nm)**	***m/z***	**Molecular formula**
Lys-AA	198.4	217.1490	C_9_H_19_N_3_O_3_
Trp-AA	217.9	275.1337	C_14_H_17_N_3_O_3._
GABA-AA 1	193.1	174.1077	C_7_H_14_N_2_O_3_
GABA-AA 2	193.4	246.1443	C_10_H_19_N_3_O_4._
Gly-AA 1	194.9	146.0764	C_5_H_10_N_2_O_3_
Gly-AA 2	192.9	217.1135	C_8_H_15_N_3_O_4_

In the lysine-acrylamide system, Lys-AA (MW = 146 + 71 = 217) with [M+H]^+^ at m/z 218.1499 was identified. HRMS result showed that the formula was C_9_H_19_N_3_O_3_, with the signal at m/z 142 indicating losing of [–CH_2_CH_2_CONH_2_] and [-NH_2_] (Table 4). ^1^H NMR spectrum showed one set of lysine protons (δ_H =_ 3.23, 1.67, 1.38, 2.97 and 2.53 ppm) and two methylene protons (δ_H_ = 2.88 and 2.94 ppm, δ_H_ = 1.67 ppm). The ^13^C NMR spectrum displayed two carbonyl signals (δ_C_ = 176.77 and 179.51 ppm), six methylene signals (δ_C_ = 31.19, 21.91, 39.16, 33.64, 43.14, and 26.61 ppm), and one methine (δ_C_ = 63.04 ppm). In addition, the heteronuclear multiple bond correlation (HMBC) from H-2 to C-3, C-4 and C-7, from H-4 to C-5, and from H-7 to C-2 and C-8 suggested that this adduct was formed by nucleophilic addition of amine group of lysine and double bond of acrylamide. Adams et al. (28) also detected the substance with a molecular weight of 217 in the lysine-acrylamide reacting system at pH 11.75 and 180°C for 2 h, but they did not share the spectrum.

**Table 4:** NMR spectra data of Lys-AA measured in D_2_O.

**HMBC Correlations**	**No**.	**δ_C_ (ppm)**	**δ_H_ (ppm)**	**Structural formula**	**Fragment ion structure**
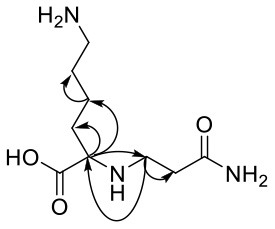	1	176.77		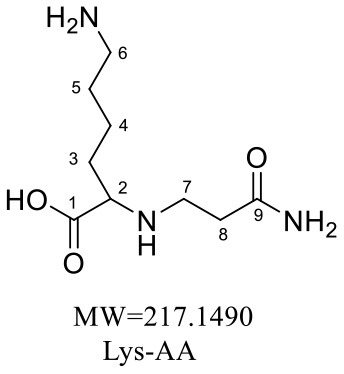	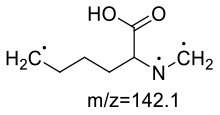
	2	63.04	3.23(dd, *J* = 7.7, 5.4 Hz, 1H)		
	3	31.19	1.67 (m, 2H)		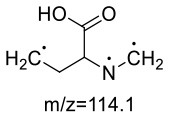
	4	21.91	1.38 (m, 2H)		
	5	39.16	2.97(m, 2H)		
	6	26.61	1.67 (m, 2H)		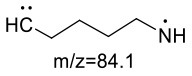
	7	43.14	2.94, 2.88(m, 2H)		
	8	33.64	2.53(td, *J* = 6.8, 3.3 Hz, 2H)		
	9	179.51			

Trp-AA (MW = 204 + 71 = 275) was detected as [M+H]^+^ m/z 276.1337 with formula of C_14_H_17_N_3_O_3_ by HRMS. The ion at m/z 230 and m/z 188 suggested the loss of [–COOH] and [–NHCH_2_CH_2_CONH_2_] (Table 5). The ^1^H NMR spectrum showed one set of tryptophan protons (3.41, 3.01, 3.19, 7.20, 7.32, 7.05, 6.96 and 7.56 ppm), and two methylene protons (δ_H_ = 2.73 and 2.91 ppm, δ_H_ = 2.31 ppm). The ^13^C NMR spectrum displayed two carbonyl signals (δ_C_ = 172.03 and 173.14 ppm), eight signals of C=C carbon (δ_C_ = 110.25, 124.26, 136.66, 111.75, 121.35, 118.74, 118.93 and 127.78 ppm), three methylene signals (δ_C_ = 43.64, 32.91 and 27.51 ppm), and one methine (δ_C_ = 62.78 ppm). Moreover, the HMBC correlations from H-2 to C-3, C-6 and C-7, from H-3 to C-4 and C-5, and from H-6 to C-8, which suggested this adduct was formed by nucleophilic addition between amine group of tryptophan and double bond of acrylamide (29).

**Table 5:** NMR spectra data of Trp-AA measured in DMSO-d_6_.

**HMBC correlations**	**No**.	**δ_C_ (ppm)**	**δ_H_ (ppm) (multiplicity)**	**Structural formula**	**Fragment ion structure**
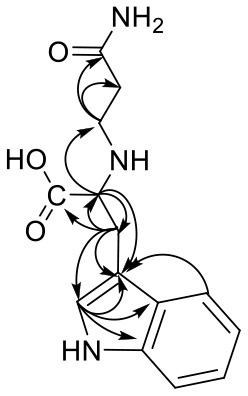	1	172.03		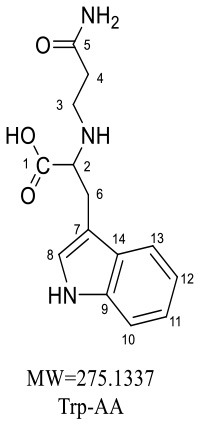	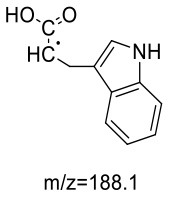
	2	62.78	3.41(t, *J* = 12.9 Hz, 1H)		
	2 -NH-		7.49(s,1H)		
	3	43.64	2.73(dt, *J* = 12.4, 6.8 Hz, 1H) 2.91(dt*,J* = 13.0, 6.8 Hz, 1H)		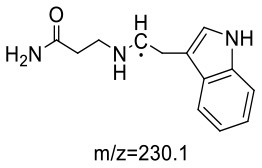
	4	32.91	2.31(td, *J* = 6.9, 3.7 Hz, 2H)		
	5	173.14			
	6	27.51	3.01(dd, *J* = 15.0, 7.4 Hz, 1H) 3.19(dd, *J* = 15.0, 5.3 Hz, 1H)		
	7	110.25			
	8	124.26	7.20(d, *J* = 2.3 Hz, 1H)		
	8 -NH-		10.86(s,1H)		
	9	136.66			
	10	111.75	7.32(d, *J* = 8.1 Hz, 1H)		
	11	121.35	7.05(m,1H)		
	12	118.74	6.96(t, *J* = 7.4 Hz, 1H)		
	13	118.93	7.56(d, *J* = 7.9 Hz, 1H)		
	14	127.78			

For the GABA-AA reaction system, two adducts, namely, GABA-AA 1 and GABA-AA 2, were separated and analyzed. GABA-AA 1 was determined as C_7_H_14_N_2_O_3_ with [M+H]^+^ m/z 175.1077, which was the total of molecular weight of γ-aminobutyric acid (MW = 103) and acrylamide (MW = 71). After losing [-CH_2_COOH] and [-NH_2_], the fragment ion m/z 98 was detected (Table 6). The ^1^H NMR spectrum showed one set of γ-aminobutyric acid protons (δ_H_ = 2.31, 1.92 and 3.09 ppm) and two methylene protons (δ_H_ = 3.32 and 2.74 ppm). The ^13^C NMR spectrum displayed two carbonyl signals (δ_C_ = 181.34 and 174.58 ppm) and five methylene signals (δ_C_ = 34.47, 22.26, 47.56, 43.21 and 30.58 ppm). In addition, the HMBC correlations from H-4 to C-2, C-3 and C-5, from H-5 to C-4, C-6 and C-7, and from H-3 to C-2 and C-4, which suggested that GABA-AA 1 was formed by nucleophilic addition between amine group of γ-aminobutyric acid and double bond of acrylamide.

**Table 6:** NMR spectra data of GABA-AA 1 measured in D_2_O.

**HMBC correlations**	**No**.	**δ_C_ (ppm)**	**δ_H_ (ppm)**	**Structural formula**	**Fragment ion structure**
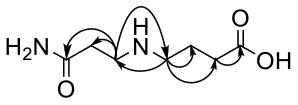	1	181.34		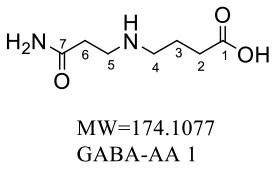	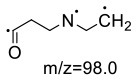
	2	34.47	2.31 t, (J= 7.0Hz,2H)		
	3	22.26	1.92 (m, J = 7.4Hz,2H)		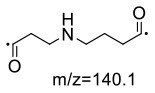
	4	47.56	3.09 (t, J = 7.6Hz,2H)		
	5	43.21	3.32 (t, J = 6.8Hz,2H)		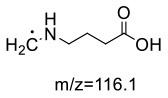
	6	30.58	2.74 (t,J = 6.8Hz,2H)		
	7	174.58			

The HRMS of GABA-AA 2 exhibited a peak at m/z 246.1443 [M+H]^+^ indicating a molecular formula of C_10_H_19_N_3_O_4_, which just had one more molecular weight of acrylamide than GABA-AA 1 (174 + 71 = 245), indicating that GABA-AA 2 was a di-AA adduct of GABA. The base peak at m/z 187 was referred to as the loss of [-CH_2_CONH_2_] from the molecular ion (Table 7). Comparison of the ^1^H and ^13^C NMR spectral data of GABA-AA 2 (Table 7) with that of GABA-AA 1 (Table 6) showed a close structural relationship between these two compounds, except for the integral of two methylene protons (δ_H_ = 2.19 and 2.66 ppm) is twice as many as that of GABA-AA 1. Under the condition of slow rotation, the chemical shifts of the two hydrogen atoms combined with nitrogen atoms differed by 0.5–1 ppm, respectively δ_H_ = 6.82 and 7.41 ppm. ^13^C MNR indicated that substructure of two propionamide groups to be a symmetric framework. In addition, the HMBC correlations from H-3 to C-1, C-2 and C-4, from H-4 to C-3, C-5, C-6 and C-2', and from H-3' to C-3, C-4 and C-2'. It was speculated that GABA-AA 2 was formed by one molecular of γ-aminobutyric acid and two molecular of acrylamide.

**Table 7:** NMR spectra data of GABA-AA 2 measured in DMSO-d_6_.

**HMBC correlations**	**No**.	**δ_C_ (ppm)**	**δ_H_ (ppm)**	**Structural formula**	**Fragment ion structure**
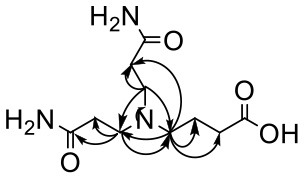	1/1'	173.93		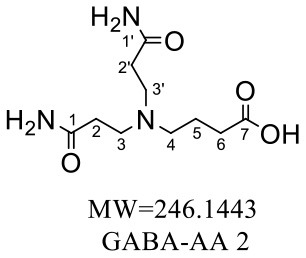	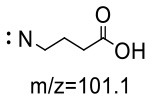
	1/1' -NH_2_		6.82 (s, 1H) 7.42 (s, 1H)		
	2/2'	32.65	2.19 (t, *J* = 7.4 Hz, 2H)		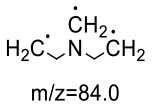
	3/3'	49.38	2.66 (t, *J* = 7.1 Hz, 2H)		
	4	52.42	2.41 (t, *J* = 7.1 Hz, 2H)		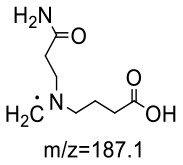
	5	22.20	1.58 (m, *J* = 7.1 Hz, 2H)		
	6	32.65	2.16 (t, 1H)		
	7	175.52			

The formation mechanism of two GABA-AA adducts was identical to Gly-AA adducts. For Gly-AA 1, the appearance of fragment ions m/z 101 and m/z 88 indicated the loss of [–COOH], [–CONH_2_] and [–CH_2_]. For Gly-AA 2, the fragment m/z 159 indicated the loss of [–CH_2_CONH_2_] and m/z 130 for losing two molecules of [–CH_2_CONH_2_] (Tables 8, 9). The ^1^H MNR spectrum of Gly-AA 1 showed three methylene protons (δ_H_ = 3.66, 3.35 and 2.77 ppm). The ^13^C NMR spectrum displayed two carbonyl signals (δ_C_ = 171.12 and 174.75 ppm), and three methylene signals (δ_C_ = 49.27, 43.35 and 30.40 ppm). Comparison of the ^1^H and ^13^C NMR spectral data of Gly-AA 2 (Table 9) with that of Gly-AA 1 (Table 8) showed a close structural relationship between these two compounds, except for the integral of two methylene protons (δ_H_ = 3.55 and 2.84 ppm) is twice as many as that of Gly-AA 1. On the basis of the above analysis, Gly-AA 2 could be proposed to be a di-AA adduct of Glycine. This structure is consistent with the results reported by Liu et al. (25).

**Table 8:** NMR spectra data of Gly-AA 1 measured in D_2_O.

**No**.	**δ_C_ (ppm)**	**δ_H_ (ppm)**	**Structural formula**	**Fragment ions structure**
1	171.12		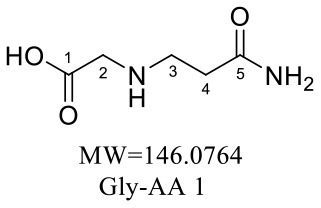	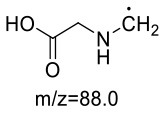
2	49.27	3.66 (d, 2H)		
3	43.35	3.35 (t, *J* = 6.7 Hz, 2H)		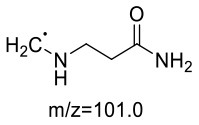
4	30.40	2.77 (t, *J* = 6.7 Hz, 2H)		
5	174.75			

**Table 9:** NMR spectra data of Gly-AA 2 measured in D_2_O.

**No**.	**δ_C_ (ppm)**	**δ_H_ (ppm)**	**Structural formula**	**Fragment ions structure**
1/1'	174.36		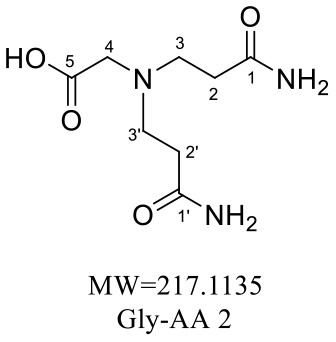	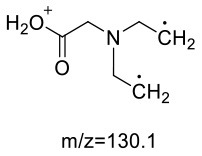
2/2'	28.78	2.84 (t, *J* = 6.8 Hz, 2H)		
3/3'	51.31	3.55 (t, *J* = 7.2 Hz, 2H)		
4	56.14	3.81 (s,2H)		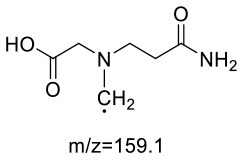
5	170.14			

**Figure 4:**
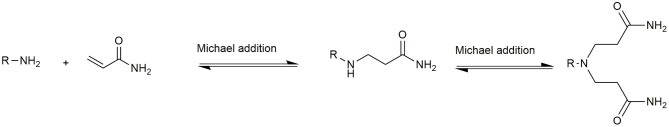
Supposed formation scheme for of amino acids-acrylamide adducts.

The above information confirmed that acrylamide reacts with the nucleophilic amino groups on amino acid side chains (27). Olefinic double bond and amide group are the two reactive sites of acrylamide. The double bond of acrylamide firstly reacts with the amino group in amino acids to form a Michael addition adduct (Figure 4). Since this adduct still has a nucleophilic group (-NH), it might attack another molecule of acrylamide to produce the dimer. Lysine and tryptophan, however, only had one major adduct with acrylamide, possibly owning to serious steric hindrance of the adduct generated in the first step, which was difficult to further react with acrylamide.

The levels of six adducts formed in baking foods were evaluated with multiple reaction monitoring (MRM) mode by mass spectrometry. Linear equation, determination coefficients, and linear range are listed in Table 10. The standards of six adducts had good linearity in a specific concentration range, and the determination coefficients were all >0.996.

**Table 10:** Standard curves of AA and six adducts.

**Additive species**	**Linear equation**	**Determination coefficients (*R*^2^)**	**LOD(μg/L)**	**LOQ(μg/L)**	**Linear range (μg/L)**
AA	Y = 405467X+5042.57	0.996	2	5	2~400
Lys-AA	Y =729807X-90291.9	0.996	5	20	5~200
Trp-AA	Y = 3708160X-3860.05	0.997	1	3	1~50
GABA-AA 1	Y = 15504500X-76457.0	0.997	3	10	3~400
GABA-AA 2	Y = 13956800X+41748.8	0.999	0.5	1	0.5~100
Gly-AA 1	Y = 2950500X+11684.9	0.998	5	20	5~250
Gly-AA 2	Y = 2526290X+11086.8	0.997	1	4	1~50

*LOD, Limit of Detection*.

*LOQ, Limit of Quantitation*.

Ten food samples were made from potatoes or cereals. Different raw materials contributed to various adduct distribution (Table 11). High levels of AA (1178.2–2260.3 μg/kg) and GABA-AA 1 (1,685–1,995 μg/kg) were detected in three brands of potato chips because uncooked potato flake was rich in free asparagine and GABA (30). GABA-AA 2 content, however, was much lower than GABA-AA1 since the acrylamide content to form a dimer in foods was less than that in model system (27). Similarly, the dimer of glycine was also far less than Gly-AA 1. Lysine adducts were found in most of food samples while the contents were below 120 μg/kg, for its content was limited in both potato and cereals. Tryptophan adduct was only found in cereal bars (22.6 μg/kg) and fried dough twists (7.3 μg/kg), which are made from oat, corn or added with sesame. Therefore, although tryptophan is thermally unstable, its adduct was found in these two food samples (31–33).

**Table 11:** The content of acrylamide and adducts in foods.

Snake food^*^	**Main composition**	**AA (μg/kg)**	**Lys-AA (μg/kg)**	**Trp-AA (μg/kg)**	**GABA-AA 1 (μg/kg)**	**GABA-AA 2 (μg/kg)**	**Gly-AA 1 (μg/kg)**	**Gly-AA 2 (μg/kg)**
Potato chips A	potato	2620.3 ± 474.6	ND	ND	1997.5 ± 155.6	ND	804.5 ± 55.2	225.6 ± 68.7
Potato chips B	potato	1178.2 ± 173.5	104.3 ± 3.3	ND	1695.0 ± 153.9	ND	482.2 ± 49.8	45.5 ± 4.2
Potato chips C	potato	1357.9 ± 21.6	120.4 ± 0.4	ND	1777.5 ± 149.8	10.0 ± 2.2	645.7 ± 14.8	16.7 ± 9.2
Brown rice Cake	brown rice	76.2 ± 13.6	53.6 ± 4.8	ND	25.0 ± 4.3	ND	231.3 ± 86.6	9.6 ± 5.3
Egg biscuit	wheat flour	42.2 ± 36.6	58.5 ± 2.1	ND	40.5 ± 4.3	ND	ND	9.8 ± 1.8
Cereal bars	oatmeal, corn flour	29.6 ± i21.1	53.3 ± 1.2	22.6 ± 12.7	78.7 ±±18.8	ND	206.8 ± 26.2	ND
Fried dough twist	wheat flour	331.4 ± 102.9	64.3 ± 9.7	7.3 ± 2.4	56.2 ± 6.5	ND	ND	15.4 ± 6.1
Cracker	wheat flour	528.0 ± 166.2	53.5 ± 1.7	ND	21.2 ± 4.3	ND	ND	1.1 ± 1
Walnut cake	wheat flour	388.4 ± 25.3	52.3 ± 2.2	ND	ND	ND	ND	ND
Millet rice crust	millet rice flour	336.4 ± 145.4	50.3 ± 2.9	ND	57.5 ± 2.2	ND	ND	ND

*ND, Not detected; the content was below the LOD presented in Table 10*.

* *: Store at room temperature and belong to ready-to-eat food*.

### GABA-AA 1 Adduct Attenuates the Cytotoxicity of Acrylamide

**Figure 5:**
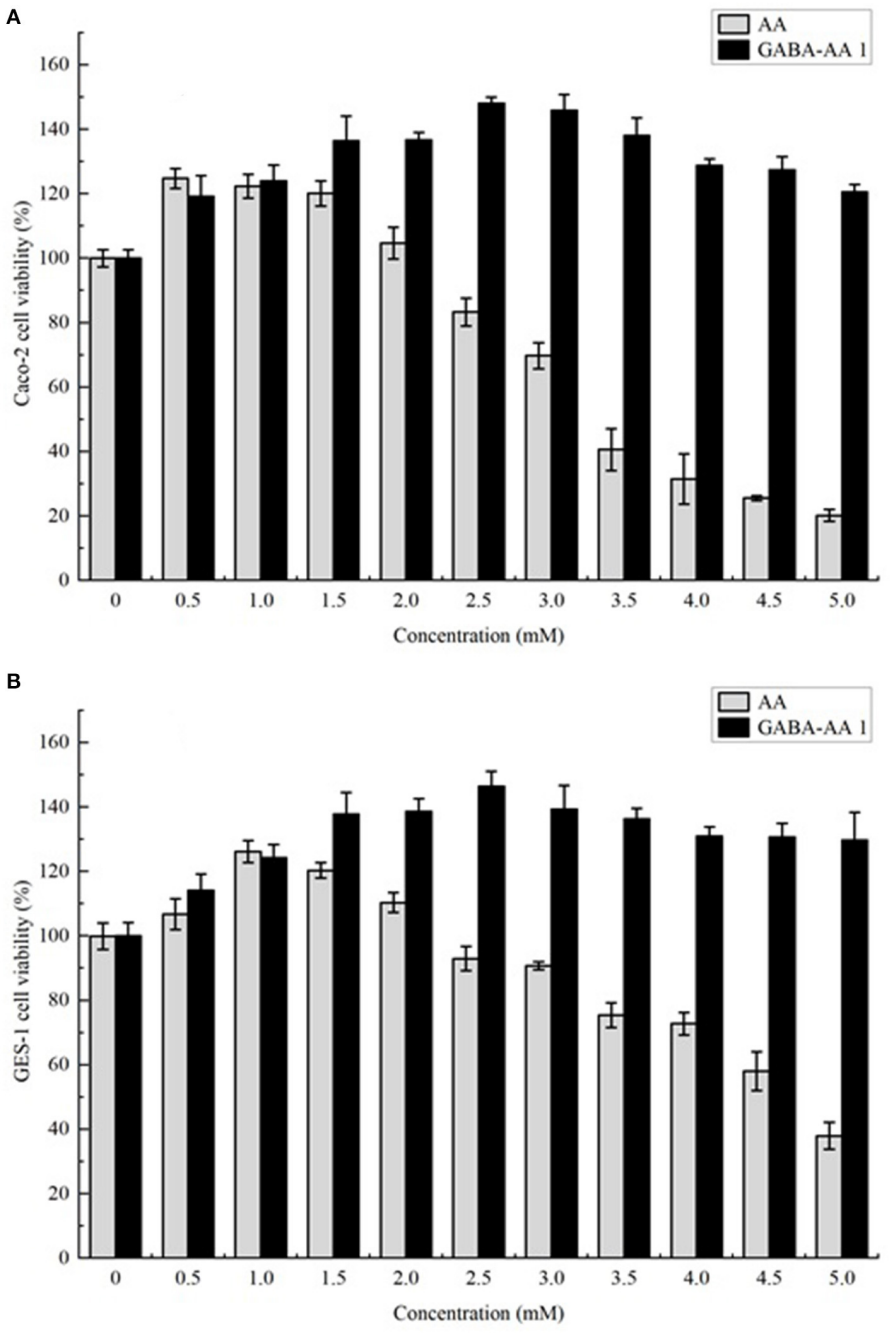
The cytotoxicity of AA and its adduct GABA-AA 1 towards Caco-2 **(A)** and GES-1 **(B)** cells.

Acrylamide is an unavoidable food contaminant in thermal processed foods. Although the appearance of adducts decreased acrylamide content in processed foods, the adducts were also inevitably exposed to humans. Whether they will pose new health risks after ingestion remains unknown. Therefore, two cell lines from gastrointestinal tract (Caco-2 cells and GES-1 cells) were applied for cytotoxicity analysis. Since the content of GABA-AA 1 was far more than other adducts, it was chosen for cell viability evaluation. As shown in Figure 5, at low concentrations, AA showed no toxic effect on cell viability in both cell lines. However, it reduced the cell viability at a concentration dependent manner over 1 mM, with IC_50_ values of 3.48 mM and 4.66 mM in GES-1 cells and Caco-2 cells, respectively. In contrast, GABA-AA 1 kept cell viability over 100% at all treated concentrations and even prompted cell growth to maximum 140%. These findings indicated that formation of GABA-AA 1 significantly decrease the cytotoxicity of AA without new health risks. As an intrinsic component of many foods, GABA may has reduced the final content of acrylamide in food processing. However, due to the limited content of GABA in food materials, acrylamide generated during food processing cannot be completely removed. Therefore, as a safe and efficient agent, GABA has the potential to be applied in food processing as additive or pretreatment, so as to mitigate the risks caused by food-derived AA.

## Conclusion

Acrylamide, a food contaminant with harmful effects on humans, can be mitigated by amino acids through forming adducts. This work analyzed the elimination rate of four amino acids and obtained six related amino acid-acrylamide adducts through chemical synthesis. Their structures were analyzed, and the formation mechanism was confirmed as Michael addition. A quantification method of these adducts was established with LC-MS/MS, and their levels in 10 commercially baking foods were determined. GABA-AA 1, the adduct formed from one molecular GABA and acrylamide, was identified at the highest levels in foods. In addition, its formation significantly reduced the cytotoxicity of acrylamide in Caco-2 cells and Ges-1 cells. This study provides the basic knowledge that amino acids may decrease acrylamide level in processed foods and lessen its deleterious effects on the body.

## Data Availability Statement

The raw data supporting the conclusions of this article will be made available by the authors, without undue reservation.

## Author Contributions

DL, FX, and KJ carried out experiments and drafted the manuscript. JZ, FL, and SO edited the manuscript. QR reviewed the submitted version. CH and JO conceived and designed this study. All authors proofread and approved the final manuscript.

## Funding

This work was financially supported by the Guangdong Natural Science Fund (no. 2018A030313064), National Natural Science Fund of China (no. 32102097), and Zhongshan Torch Modern Industrial Engineering Technology Research Institute Innovation Center (no. 2019CYY01002).

## Conflict of Interest

The authors declare that the research was conducted in the absence of any commercial or financial relationships that could be construed as a potential conflict of interest.

## Publisher's Note

All claims expressed in this article are solely those of the authors and do not necessarily represent those of their affiliated organizations, or those of the publisher, the editors and the reviewers. Any product that may be evaluated in this article, or claim that may be made by its manufacturer, is not guaranteed or endorsed by the publisher.
