# Supplementary material for: Formation and Identification of Six Amino Acid - Acrylamide Adducts and Their Cytotoxicity Toward Gastrointestinal Cell Lines
Source: Front Nutr. 2022 May 20;9:902040. doi: 10.3389/fnut.2022.902040 (PMC9167057; doi:10.3389/fnut.2022.902040)

The List of Contents

No.	Content	Page
1	Figure S1 Electrospray ionization mass spectrometry (ESI-MS) performed on $[M+H]^+$ ions of the adducts (indicated by the arrows) in Lysine-AA (A) Tryptophan-AA (B), GABA-AA (C, D) and Glycine-AA (E, F) reaction system. AA: acrylamide.	1
2	Figure S2 Secondary mass spectrometry performed on Lys-AA (m/z =218, A), Trp-AA (m/z =276, B), GABA-AA1 (m/z =175, C), GABA-AA 2 (m/z =246, D), Gly-AA 1 (m/z =147, E) and Gly-AA 2 (m/z =218, F). Fragment ions of adducts in the figure are indicated by arrows. AA: acrylamide, Lys: lysine, Trp: tryptophan, Gly: glycine	2
3	Figure S3 HRMS spectrum of Lys-AA.	3
4	Figure S4 ^{1}H NMR spectrum of Lys-AA in D_2O.	3
5	Figure S5 ^{13}C NMR spectrum of Lys-AA in D_2O.	4
6	Figure S6 Dept 135 spectrum of Lys-AA in D_2O.	4
7	Figure S7 ^{1}H-^{1}H COSY spectrum of Lys-AA in D_2O.	5
8	Figure S8 HSQC spectrum of Lys-AA in D_2O.	5
9	Figure S9 HMBC spectrum of Lys-AA in D_2O.	6
10	Figure S10 HRMS spectrum of Trp-AA.	6
11	Figure S11 ^{1}H NMR spectrum of Trp-AA in DMSO-d_6.	7
12	Figure S12 ^{13}C NMR spectrum of Trp-AA in DMSO-d_6.	7
13	Figure S13 Dept 135 spectrum of Trp-AA in DMSO-d_6.	8
14	Figure S14 ^{1}H-^{1}H COSY spectrum of Trp-AA in DMSO-d_6.	8
15	Figure S15 HSQC spectrum of Trp-AA in DMSO-d_6.	9
16	Figure S16 HMBC spectrum of Trp-AA in DMSO-d_6.	9
17	Figure S17 HRMS spectrum of GABA-AA 1.	10
18	Figure S18 ^{1}H NMR spectrum of GABA-AA 1 in D_2O.	10
19	Figure S19 ^{13}C NMR spectrum of GABA-AA 1 in D_2O.	11
20	Figure S20 Dept 135 spectrum of GABA-AA 1 in D_2O.	11
21	Figure S21 ^{1}H-^{1}H COSY spectrum of GABA-AA 1 in D_2O.	12
22	Figure S22 HSQC spectrum of GABA-AA 1 in D_2O.	12
23	Figure S23 HMBC spectrum of GABA-AA 1 in D_2O.	13
24	Figure S24 HRMS spectrum of GABA-AA 2.	13
25	Figure S25 ^{1}H NMR spectrum of GABA-AA 2 in DMSO-d_6.	14
25	Figure S26 ^{13}C NMR spectrum of GABA-AA 2 in DMSO-d_6.	14
27	Figure S27 Dept 135 spectrum of GABA-AA 2 in DMSO-d_6.	15
28	Figure S28 ^{1}H-^{1}H COSY spectrum of GABA-AA 2 in DMSO-d_6.	15
29	Figure S29 HSQC spectrum of GABA-AA 2 in DMSO-d_6.	16
30	Figure S30 HMBC spectrum of GABA-AA 2 in DMSO-d_6.	16
31	Figure S31 HRMS spectrum of Gly-AA 1.	17
32	Figure S32 ^{1}H NMR spectrum of Gly-AA 1 in D_2O.	17
33	Figure S33 ^{13}C NMR spectrum of Gly-AA 1 in D_2O.	18
34	Figure S34 Dept 135 spectrum of Gly-AA 1 in D_2O.	18

35	Figure S35 HRMS spectrum of Gly-AA 2.	19
36	Figure S36 ^{1}H NMR spectrum of Gly-AA 2 in D_2O.	19
37	Figure S37 ^{13}C NMR spectrum of Gly-AA 2 in D_2O.	20
38	Figure S38 Dept 135 spectrum of Gly-AA 2 in D_2O	20

Figure S1 Electrospray ionization mass spectrometry (ESI-MS) performed on [M+H]+ ions of the adducts (indicated by the arrows) in Lysine-AA (A) Tryptophan-AA (B), GABA-AA (C, D) and Glycine-AA (E, F) reaction system. AA: acrylamide.

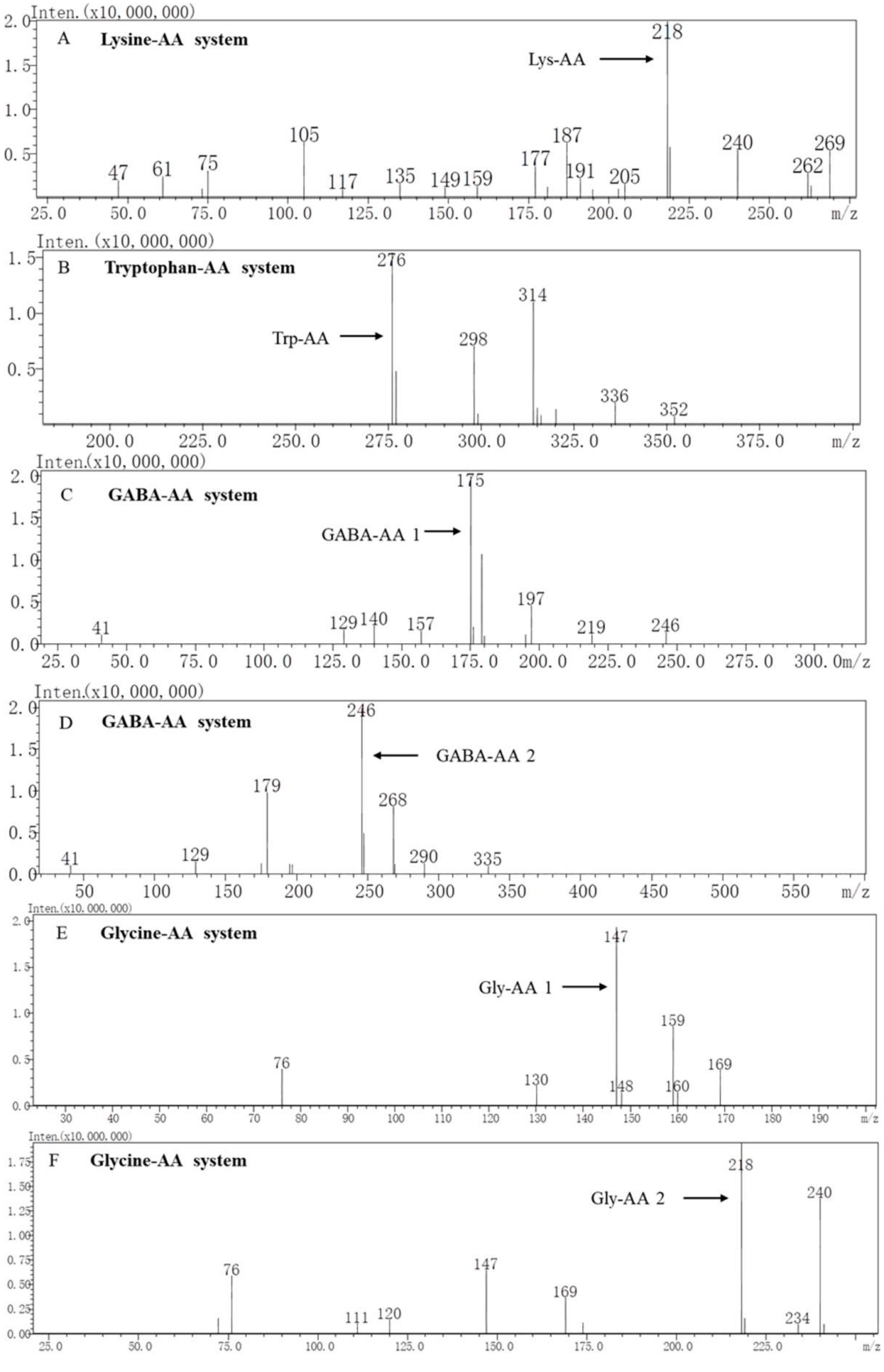

Figure S2 Secondary mass spectrometry performed on Lys-AA (m/z =218, A), Trp-AA (m/z =276, B), GABA-AA1 (m/z =175, C), GABA-AA 2 (m/z =246, D), Gly-AA 1 (m/z =147, E) and Gly-AA 2 (m/z =218, F). Fragment ions of adducts in the figure are indicated by arrows. AA: acrylamide, Lys: lysine, Trp: tryptophan, Gly: glycine.

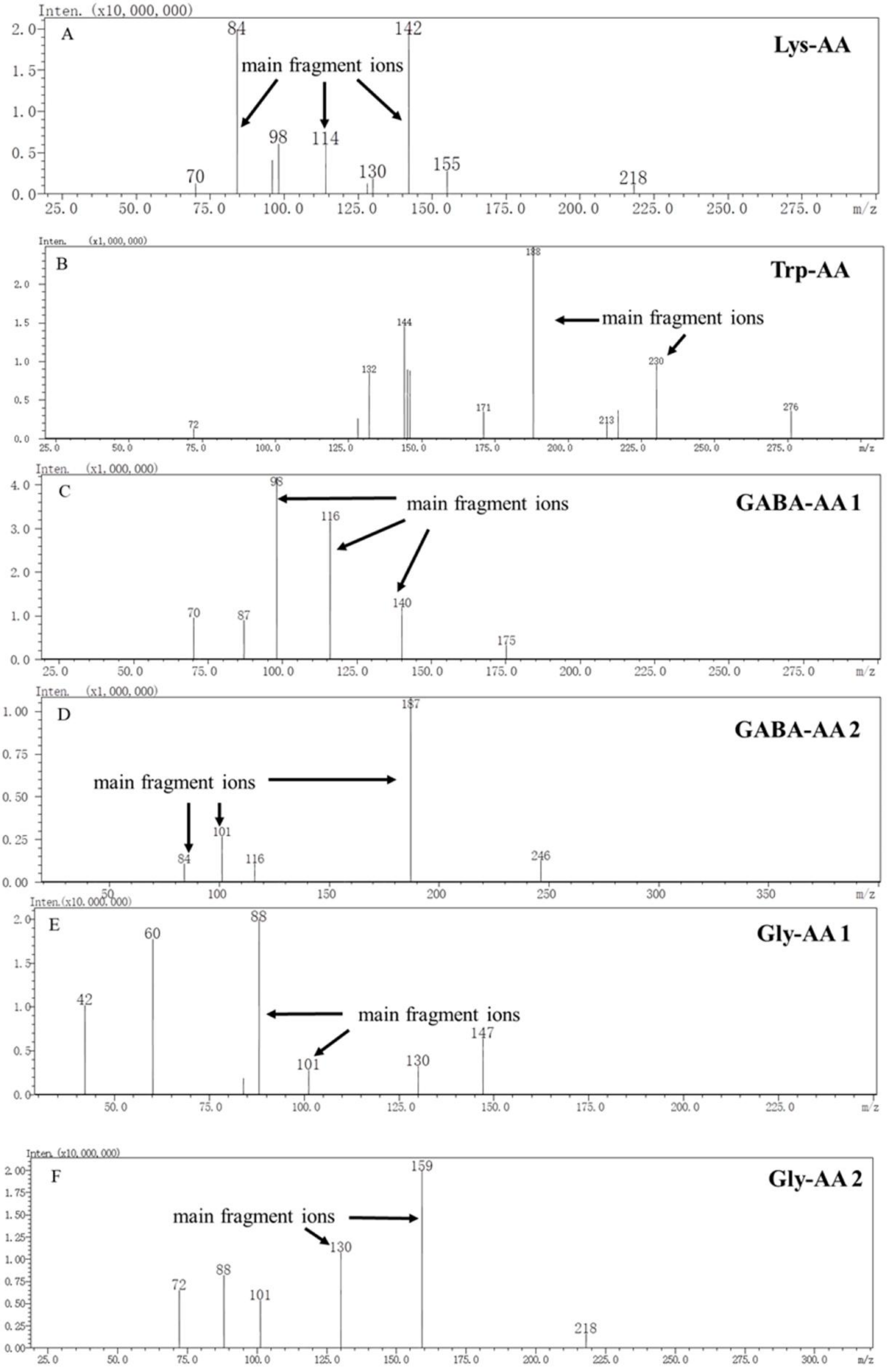

Figure S3 HRMS spectrum of Lys-AA.

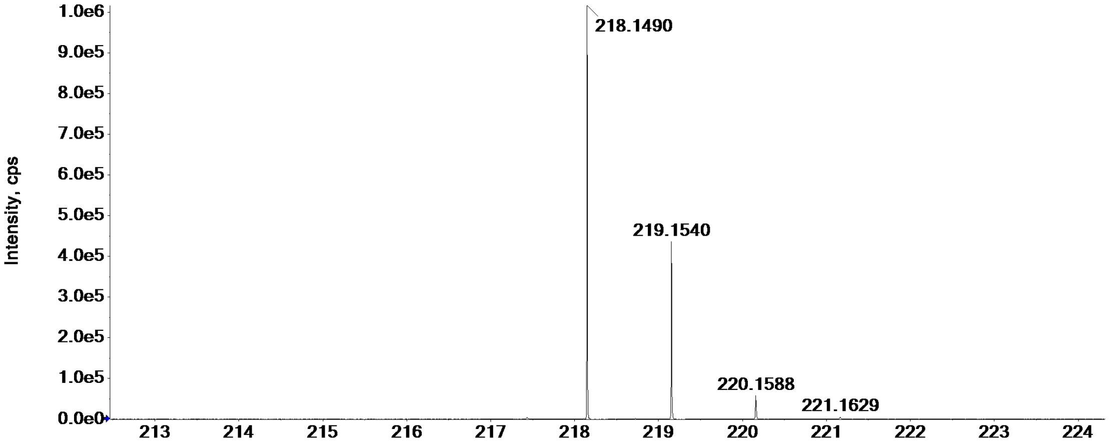

Formula	m/z	RDB	ppm
$C_9H_{16}N_2O_3$	218.1499	2.0	-4.2

Figure S4 ^{1}H NMR spectrum of Lys-AA in D_2O.

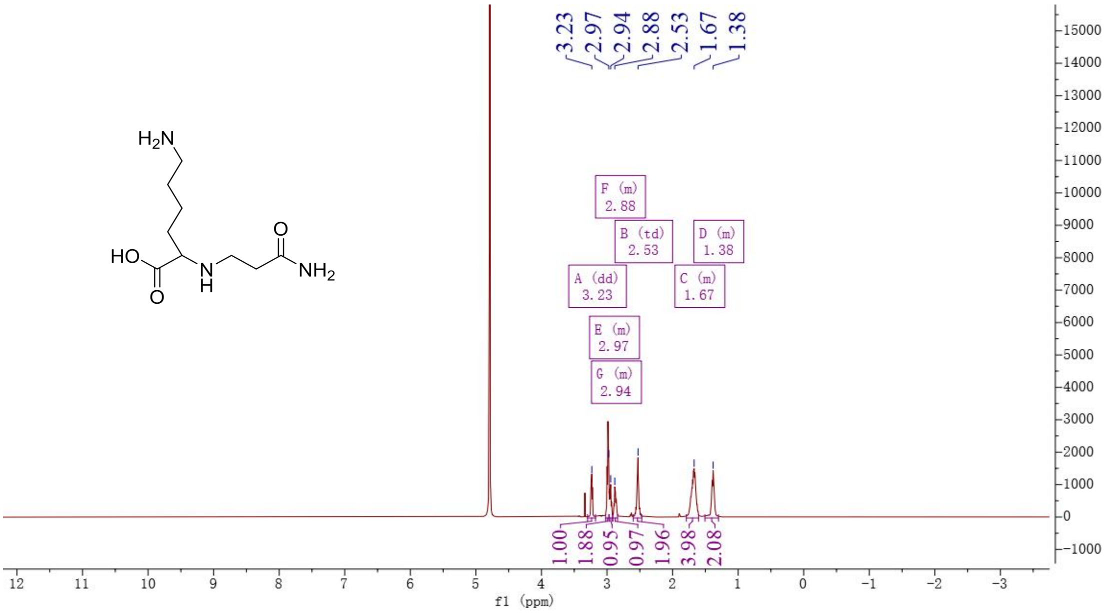

Figure S5 ^{13}C NMR spectrum of Lys-AA in D_2O.

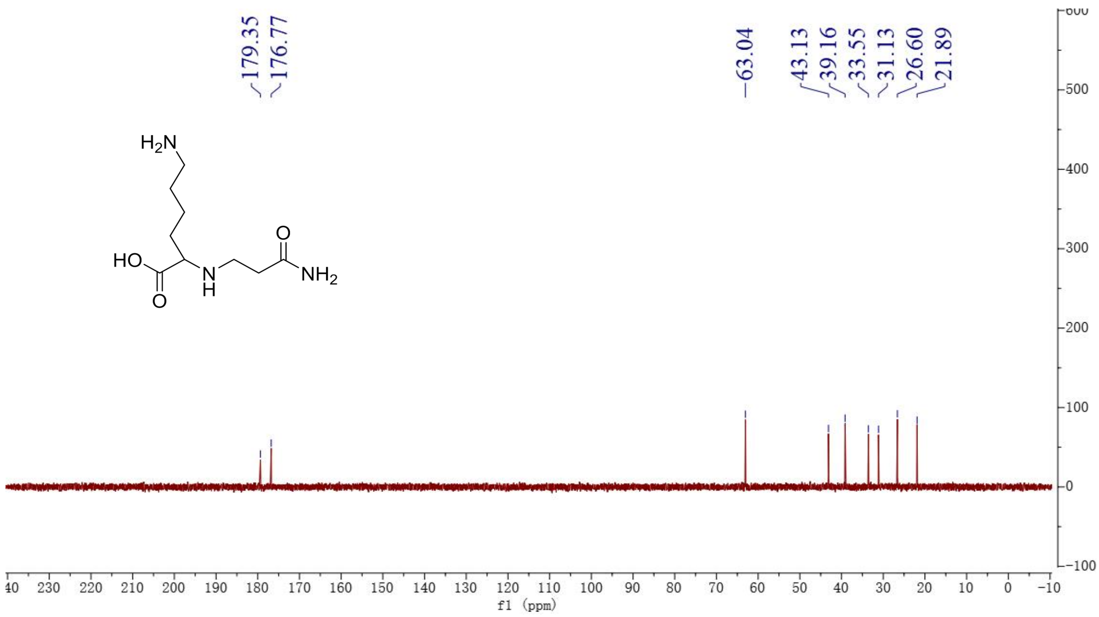

Figure S6 Dept 135 spectrum of Lys-AA in D_2O.

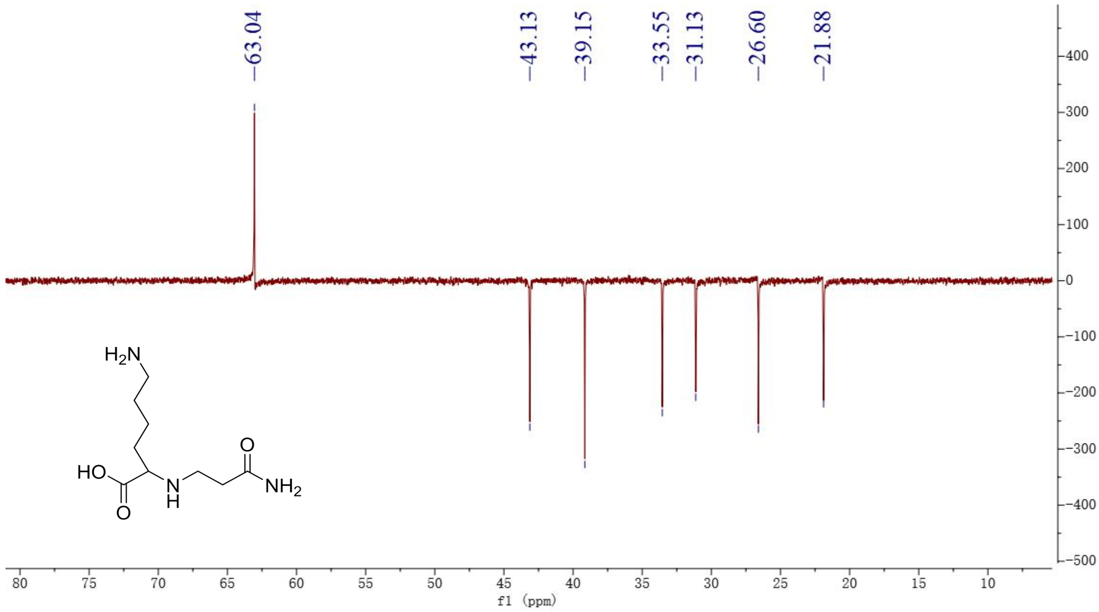

Figure S7 ^1H-^1H COSY spectrum of Lys-AA in D_2O.

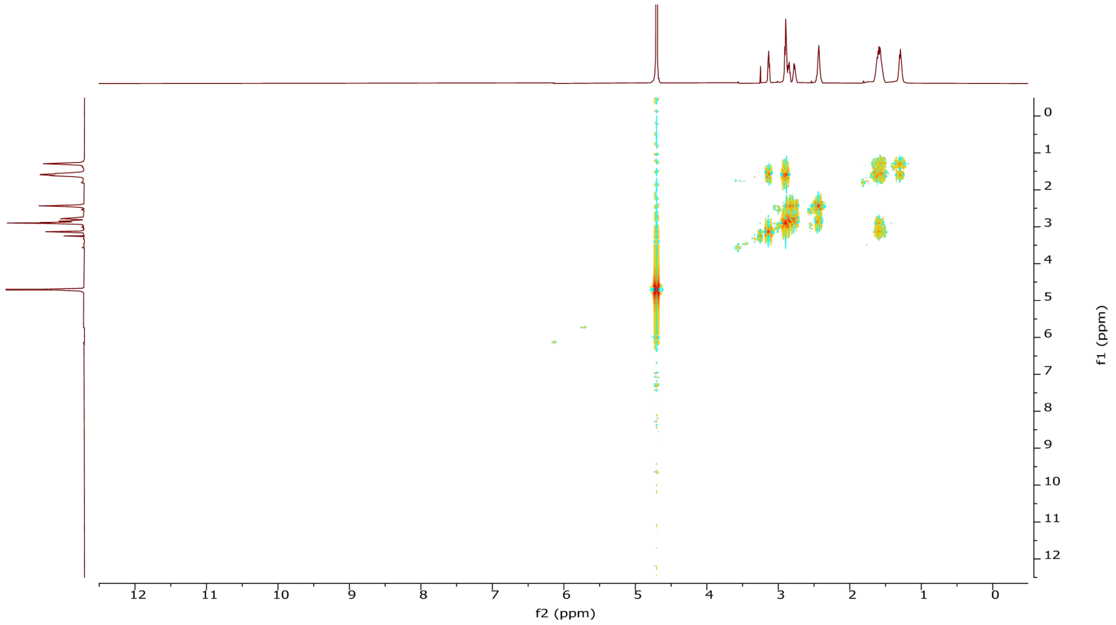

Figure S8 HSQC spectrum of Lys-AA in D_2O.

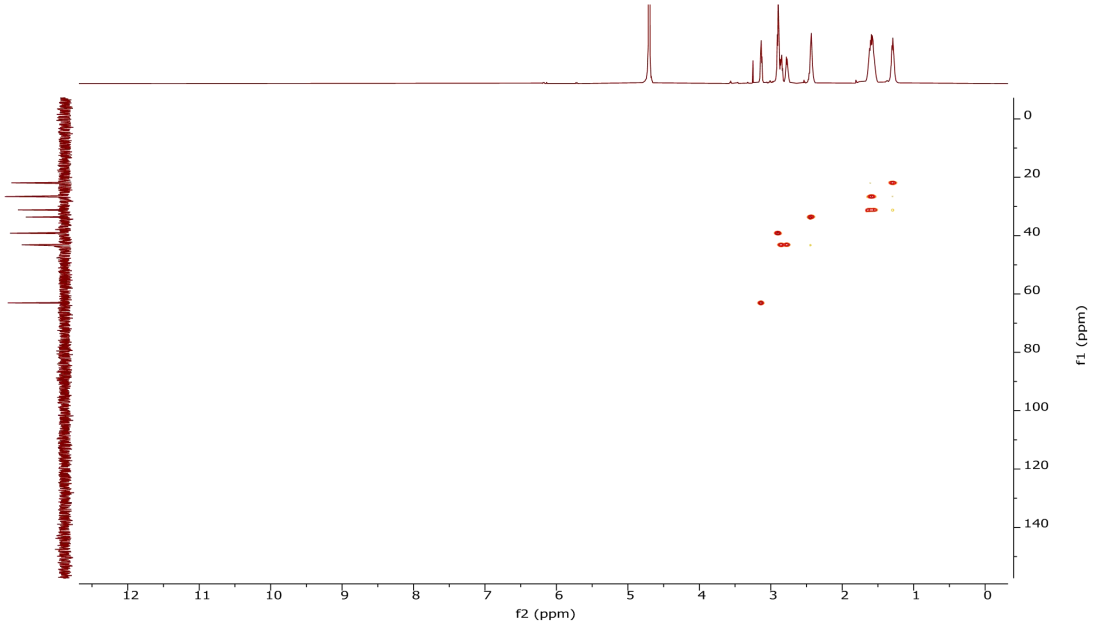

Figure S9 HMBC spectrum of Lys-AA in D_2O.

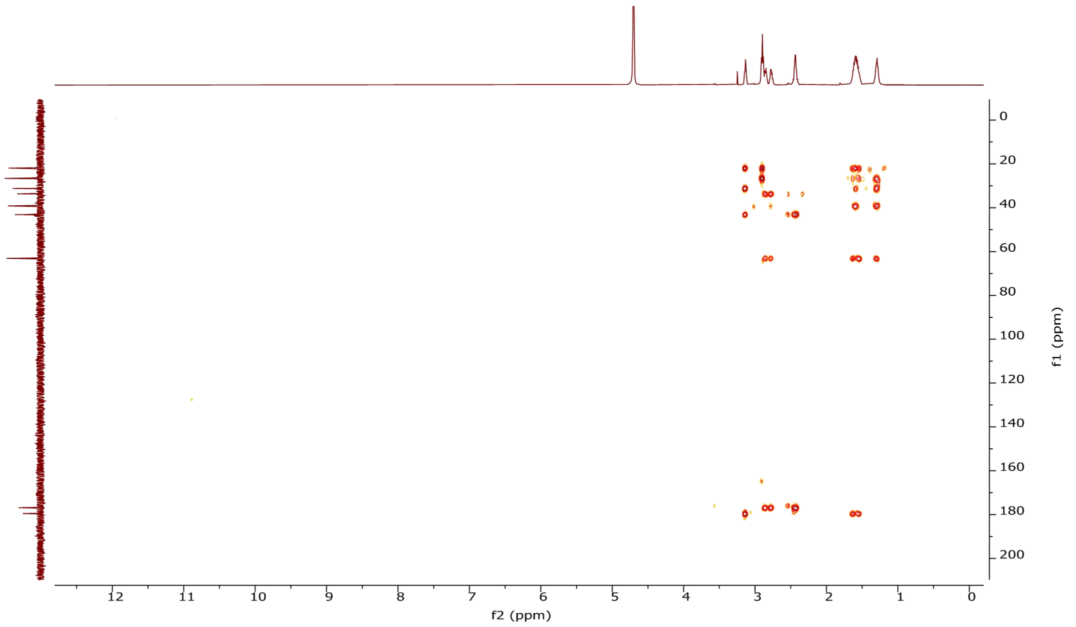

Figure S10 HRMS spectrum of Trp-AA.

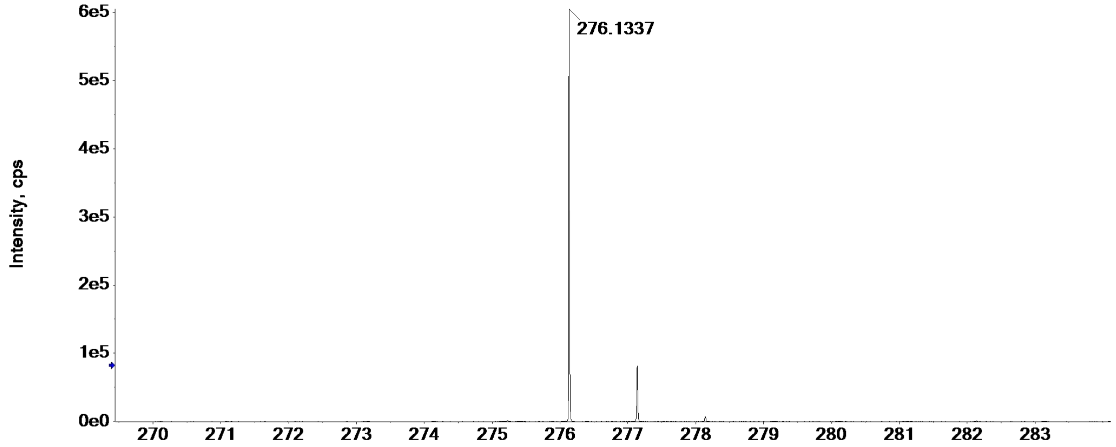

Formula	m/z	RDB	ppm
$C_{14}H_{17}N_3O_3$	276.1343	8.0	-2.1

Figure S11 ^{1}H NMR spectrum of Trp-AA in DMSO-d_6.

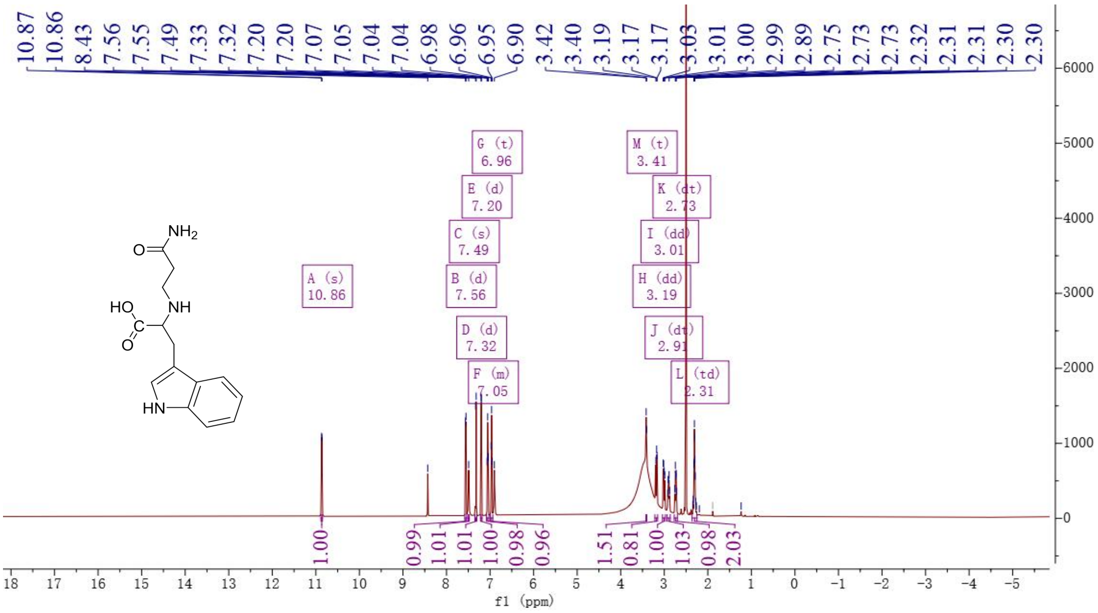

Figure S12 ^{13}C NMR spectrum of Trp-AA in DMSO-d_6.

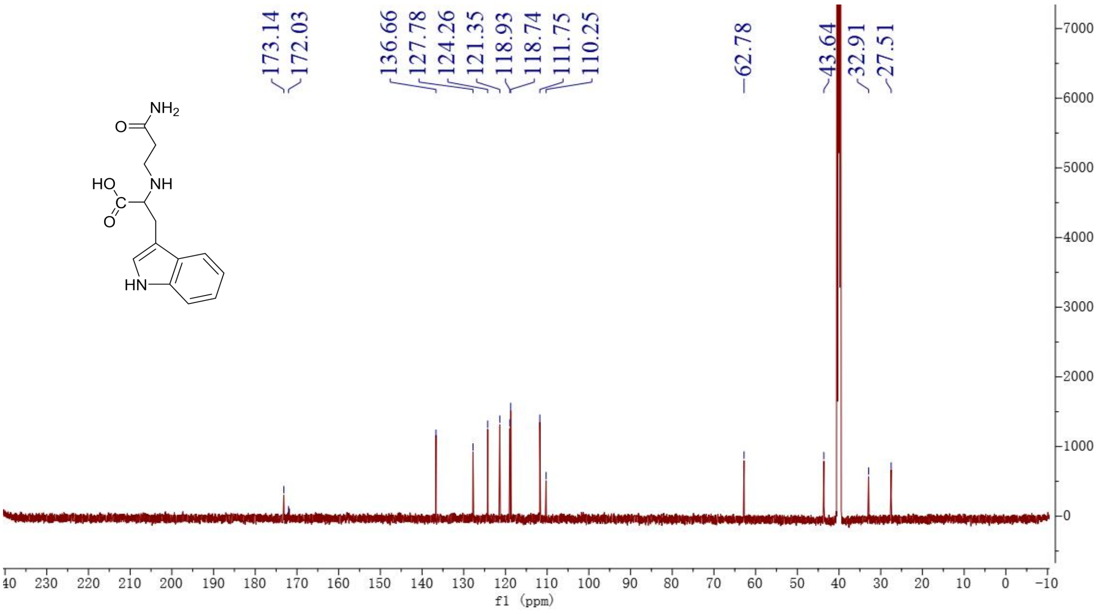

Figure S13 Dept 135 spectrum of Trp-AA in DMSO-d_6.

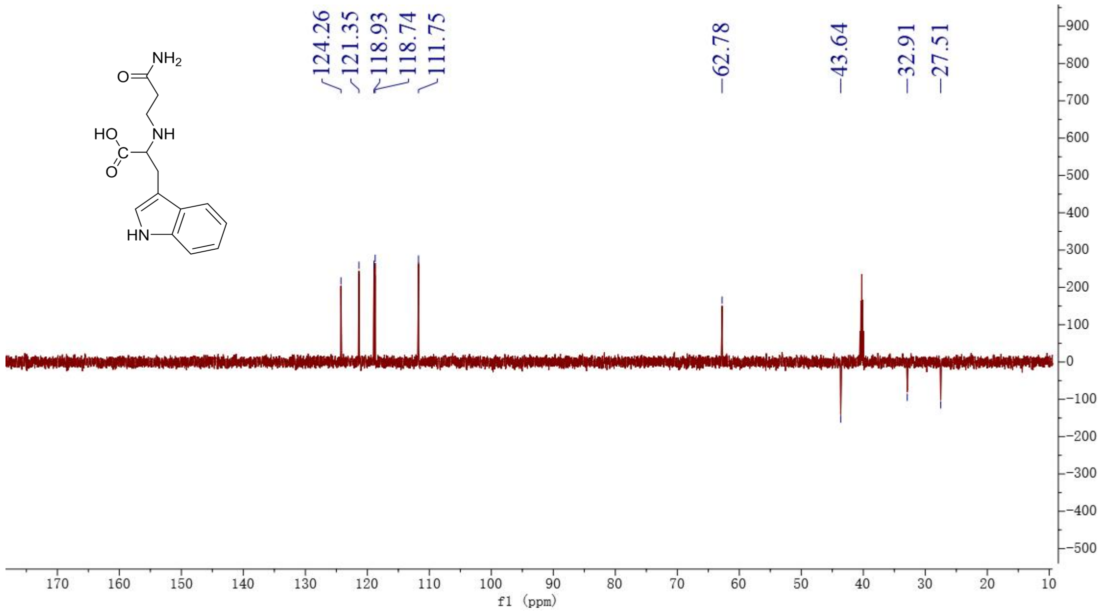

Figure S14 ^1H-^1H COSY spectrum of Trp-AA in DMSO-d_6.

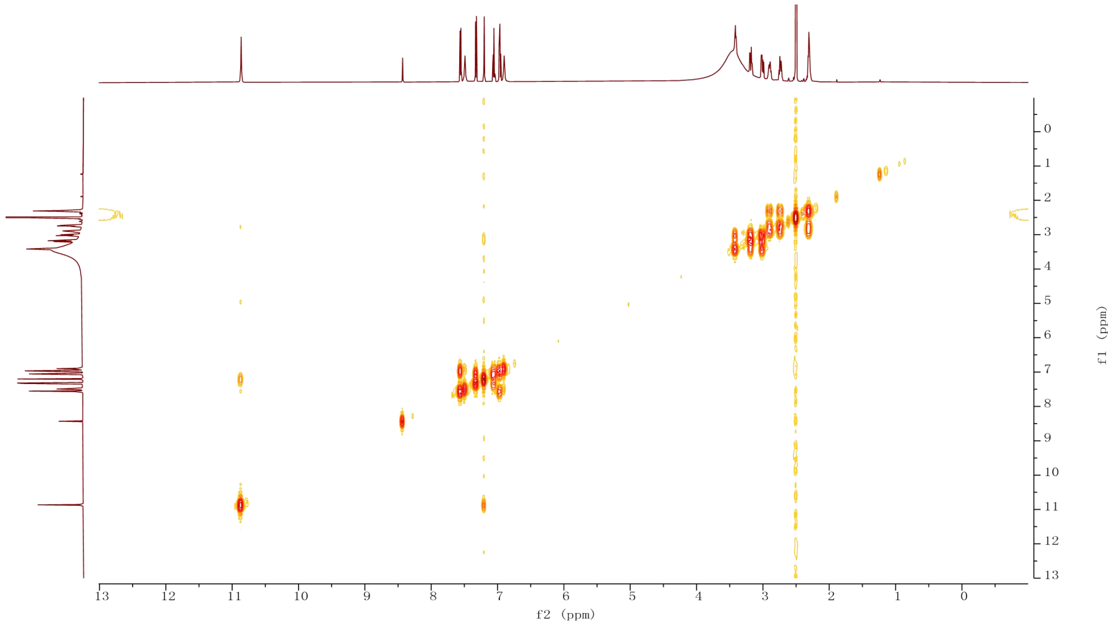

Figure S15 HSQC spectrum of Trp-AA in DMSO-d$_6$.

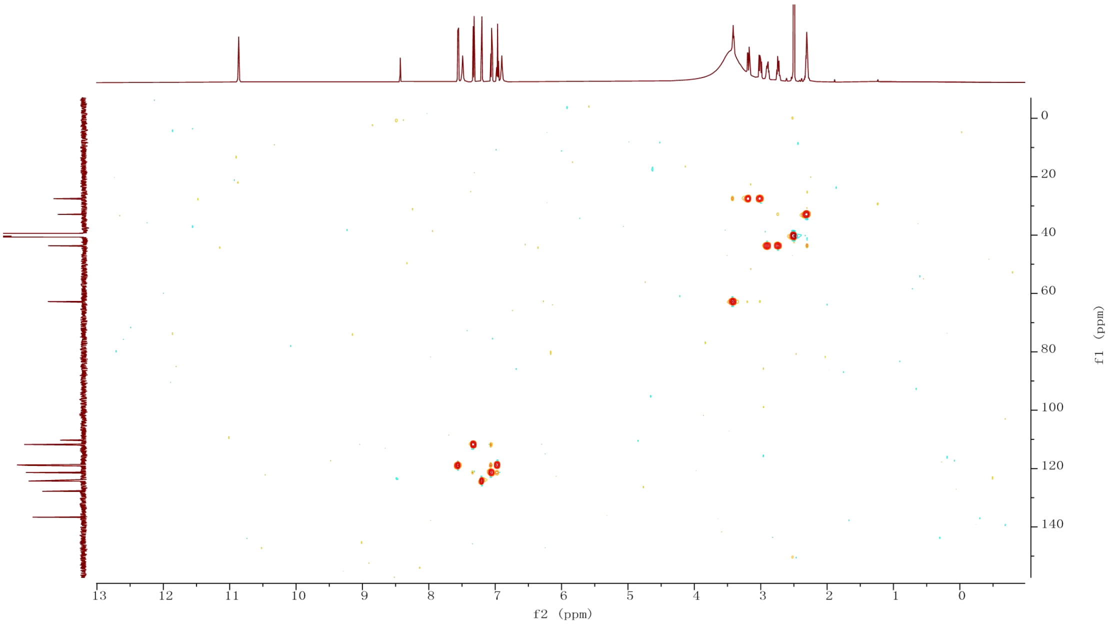

Figure S16 HMBC spectrum of Trp-AA in DMSO-d$_6$.

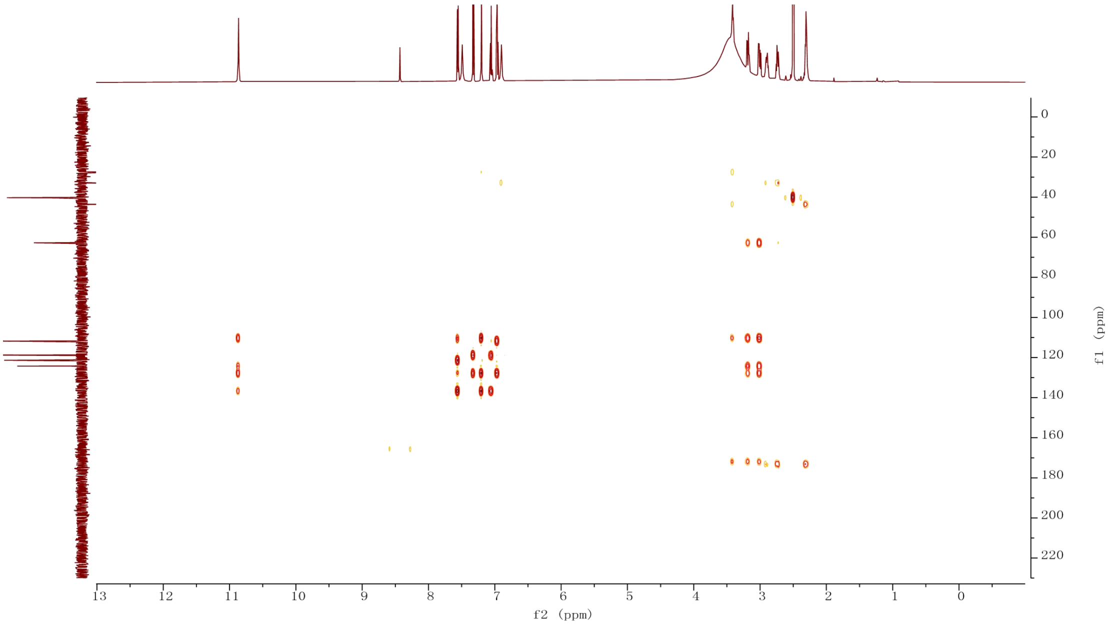

Figure S17 HRMS spectrum of GABA-AA 1.

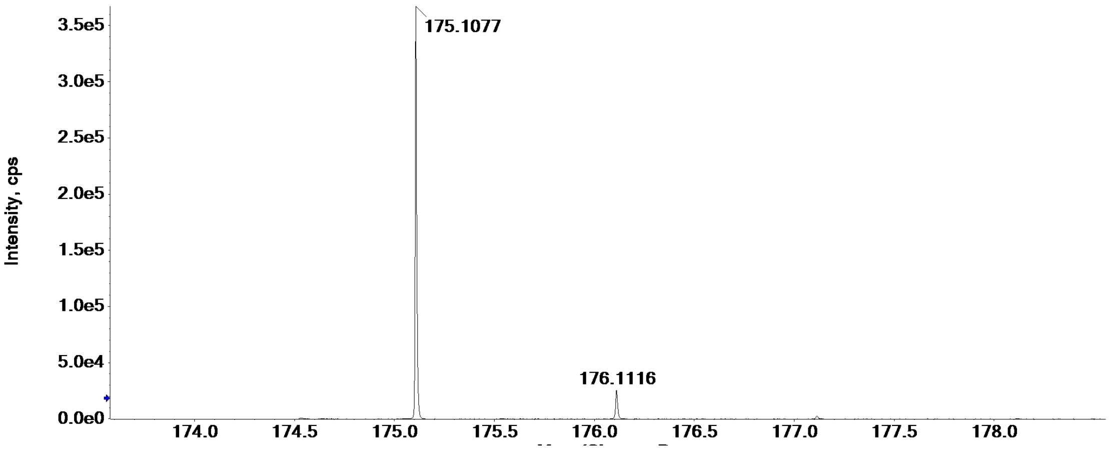

Formula	m/z	RDB	ppm
$C_7H_{14}N_2O_3$	175.1077	2.0	-0.1

Figure S18 ^{1}H NMR spectrum of GABA-AA 1 in D_2O.

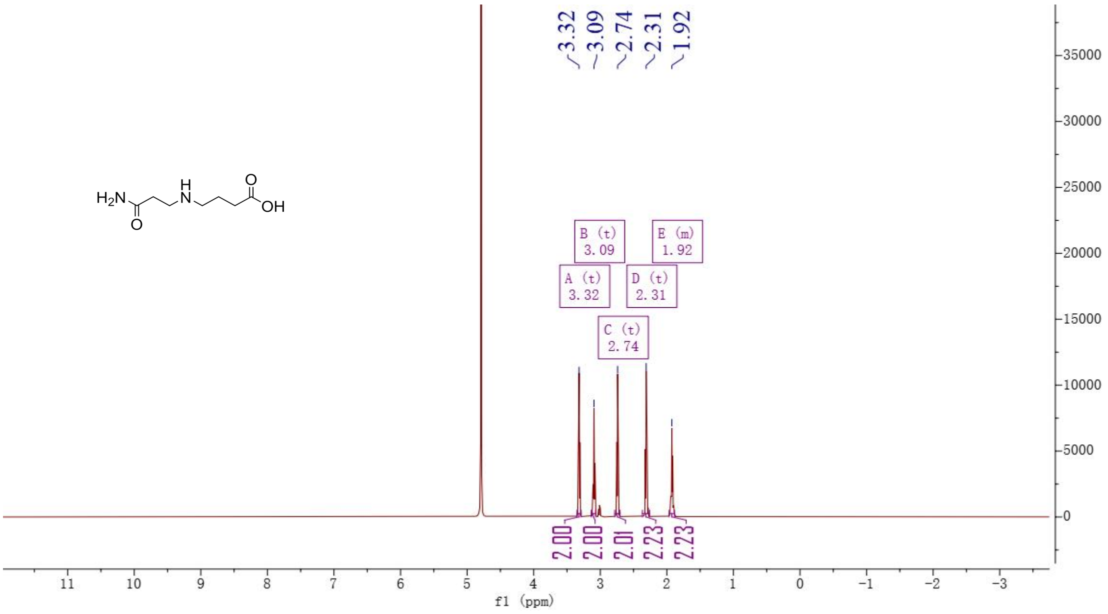

Figure S19 ^{13}C NMR spectrum of GABA-AA 1 in D_2O.

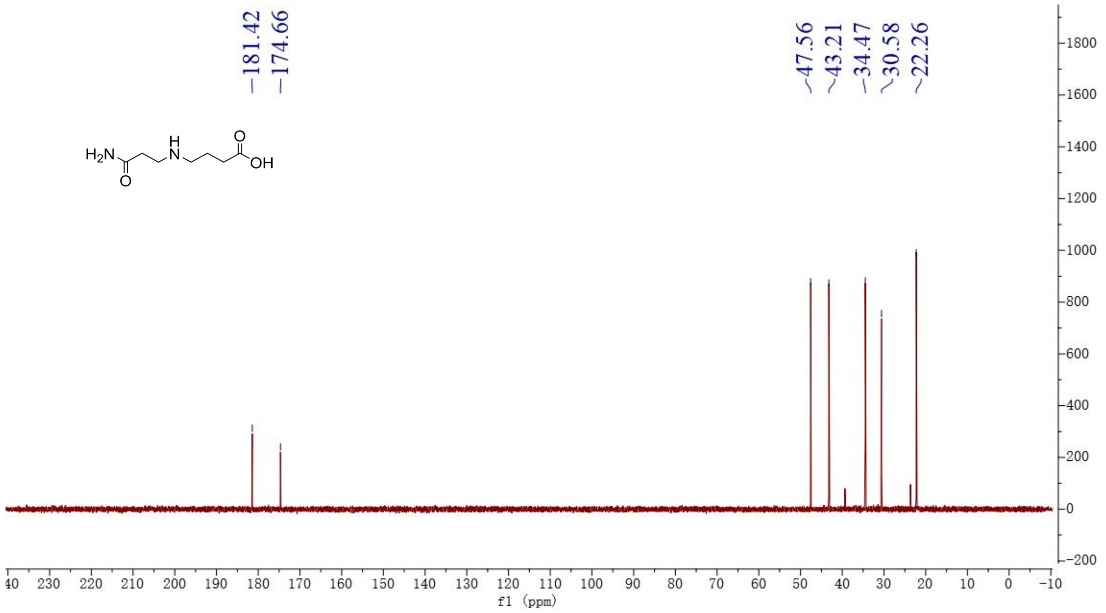

Figure S20 Dept 135 spectrum of GABA-AA 1 in D_2O.

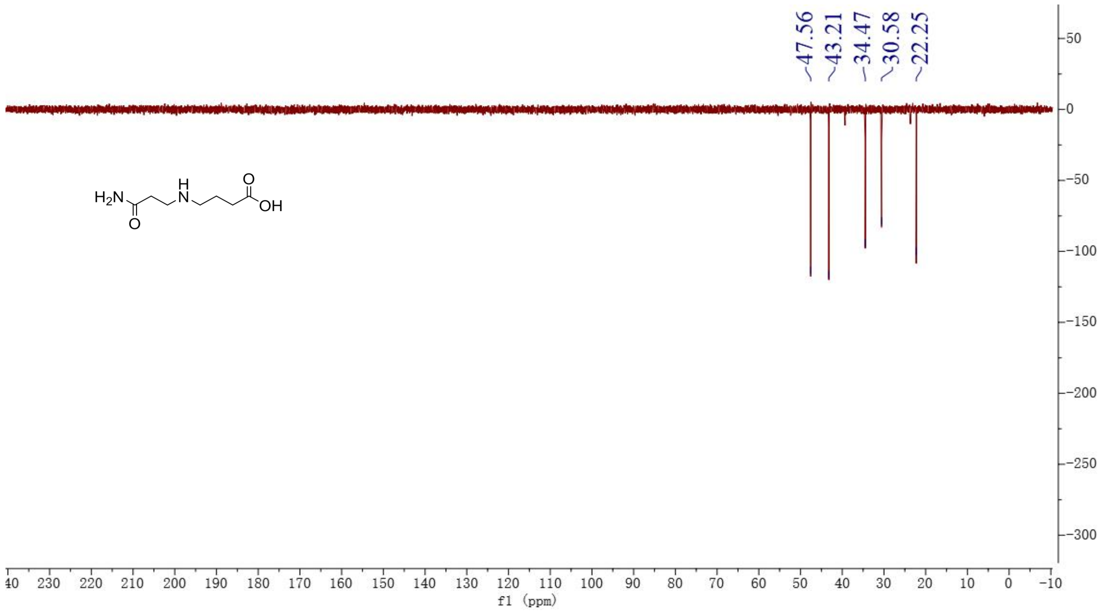

Figure S21 ^1H-^1H COSY spectrum of GABA-AA 1 in D_2O.

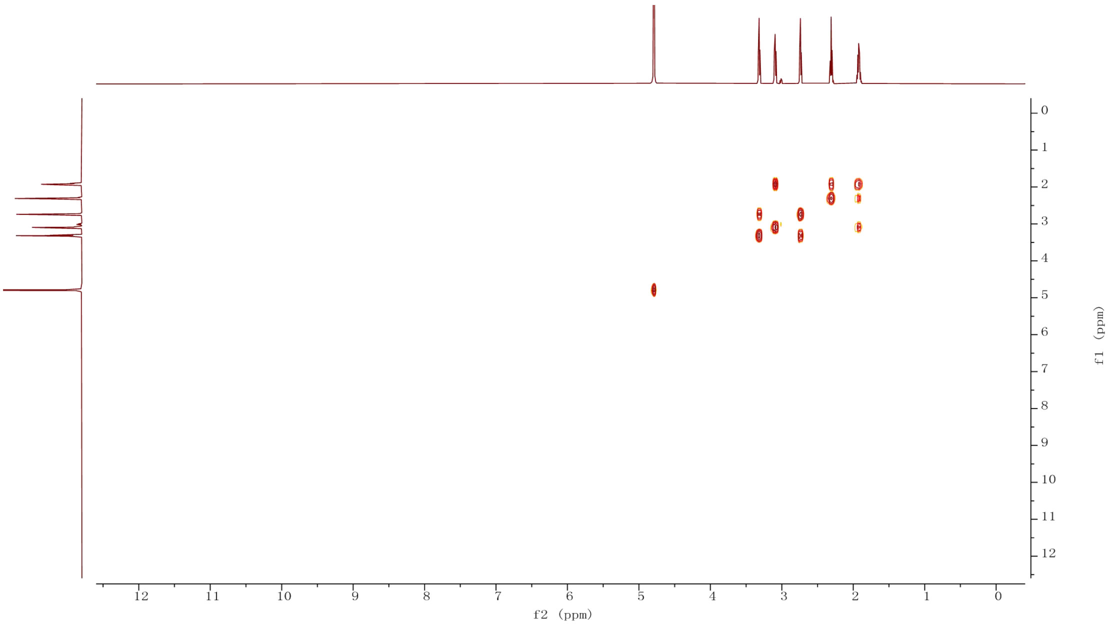

Figure S22 HSQC spectrum of GABA-AA 1 in D_2O.

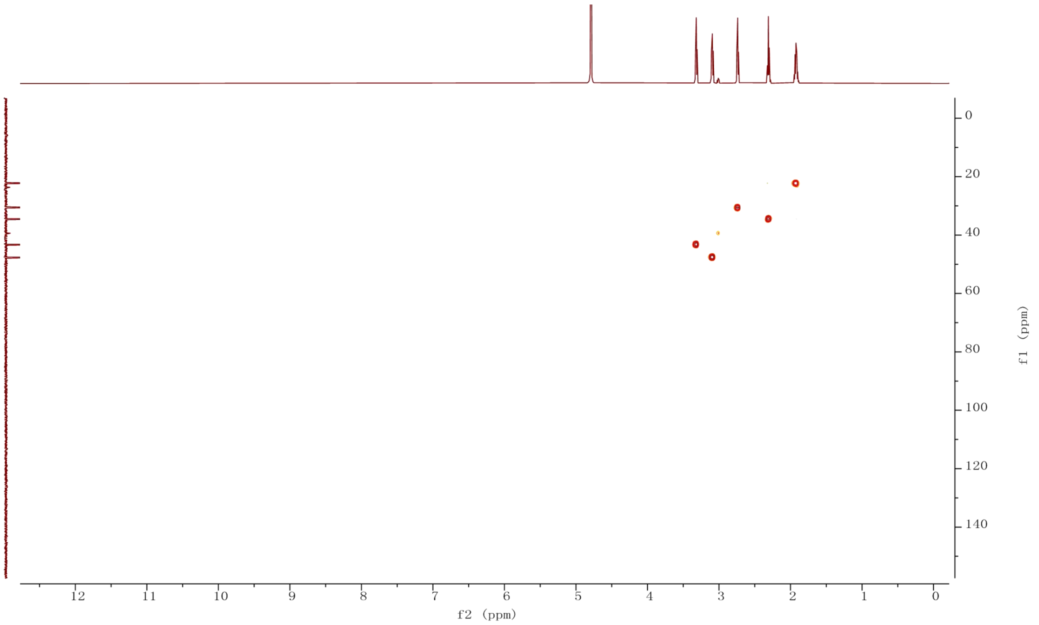

Figure S23 HMBC spectrum of GABA-AA 1 in D_2O.

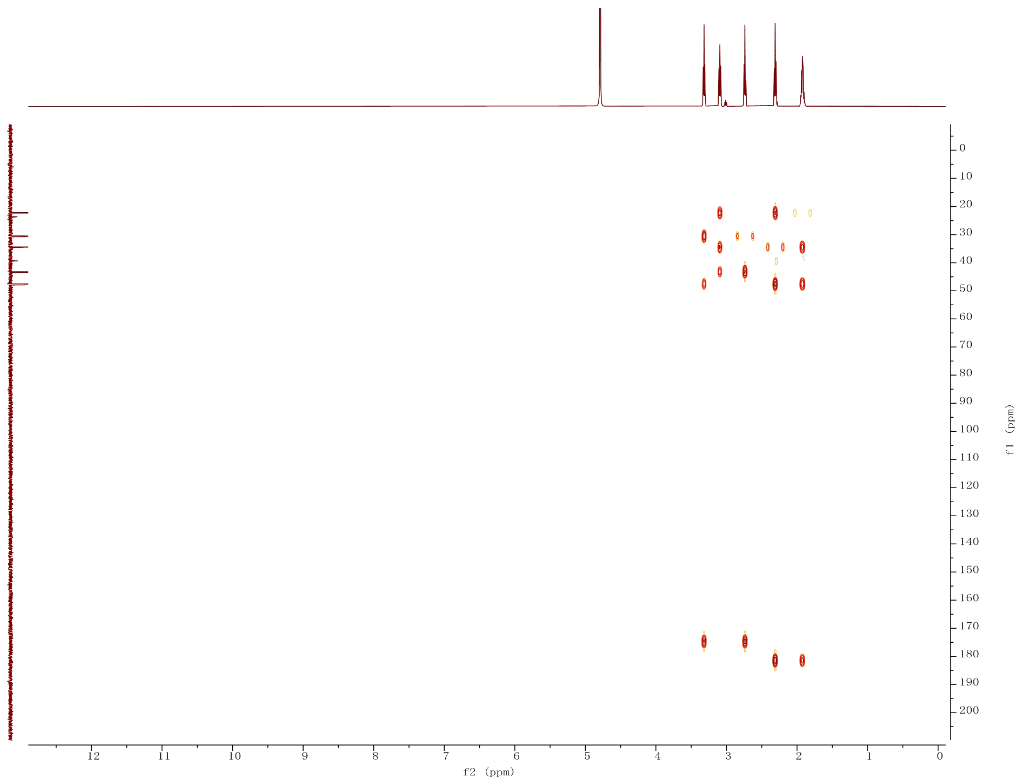

Figure S24 HRMS spectrum of GABA-AA 2.

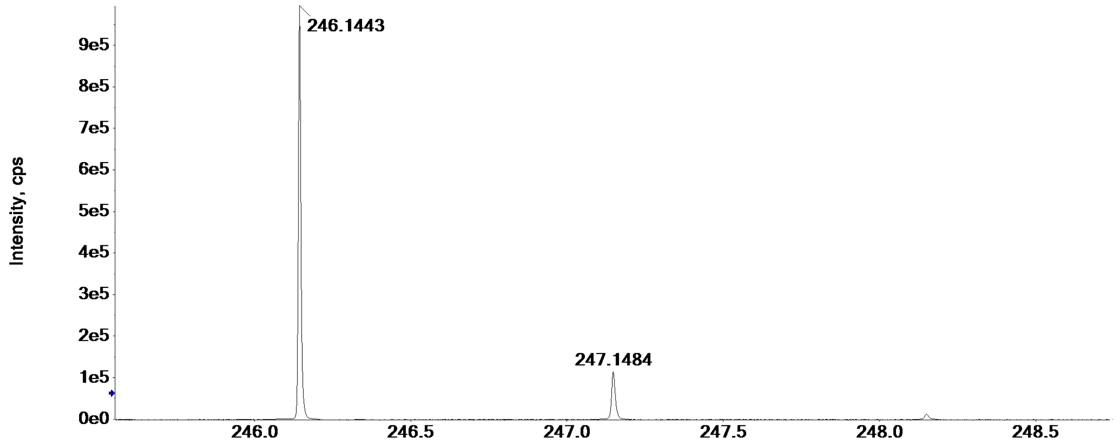

Formula	m/z	RDB	ppm
$C_{10}H_{19}N_3O_4$	246.1448	3.0	-2.2

Figure S25 ^{1}H NMR spectrum of GABA-AA 2 in DMSO-d_6.

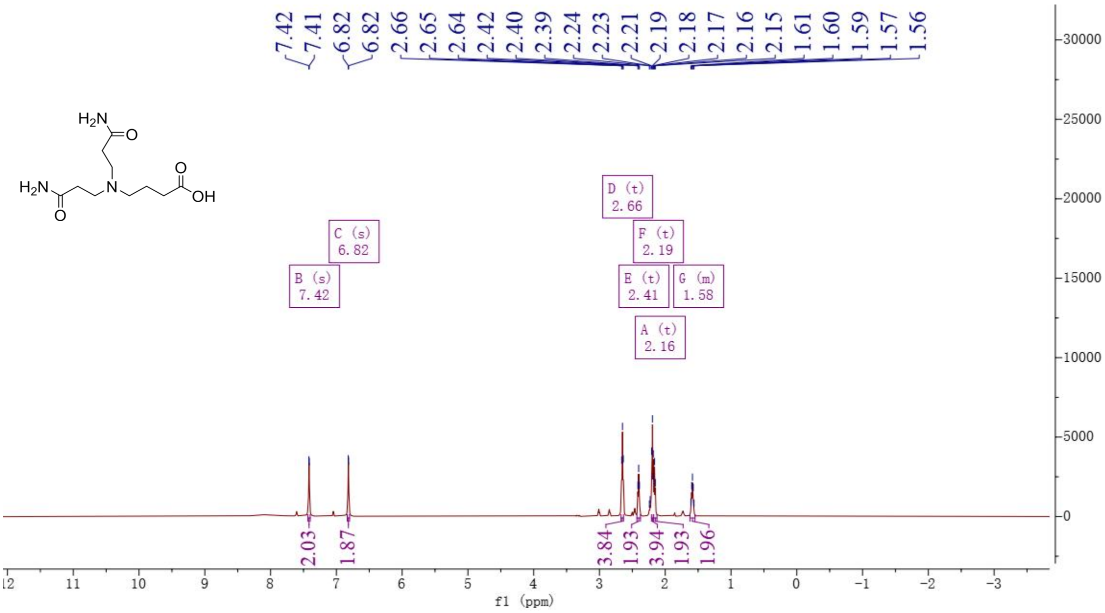

Figure S26 ^{13}C NMR spectrum of GABA-AA 2 in DMSO-d_6.

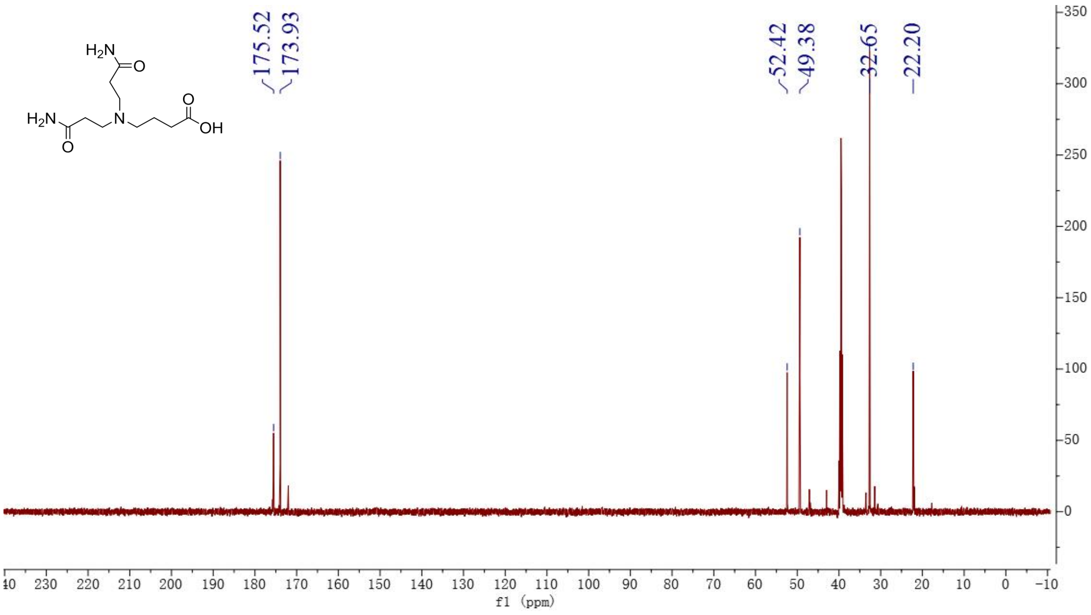

Figure S27 Dept 135 spectrum of GABA-AA 2 in DMSO-d₆.

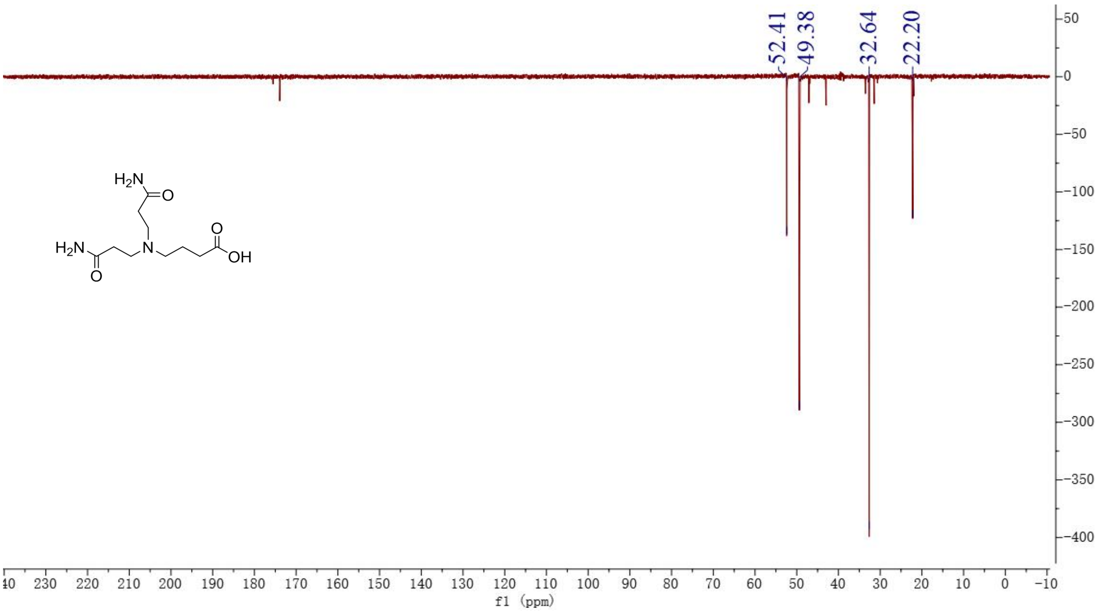

Figure S28 ^1H-^1H COSY spectrum of GABA-AA 2 in DMSO-d₆.

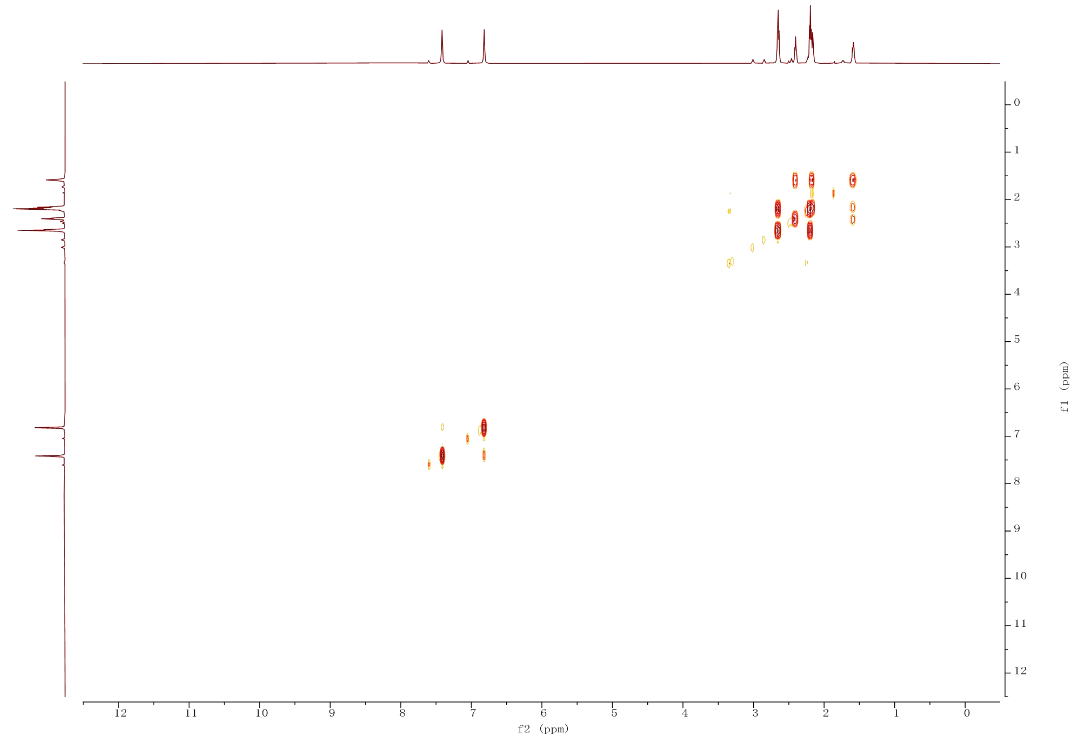

Figure S29　HSQC spectrum of GABA-AA 2 in DMSO-d$_6$.

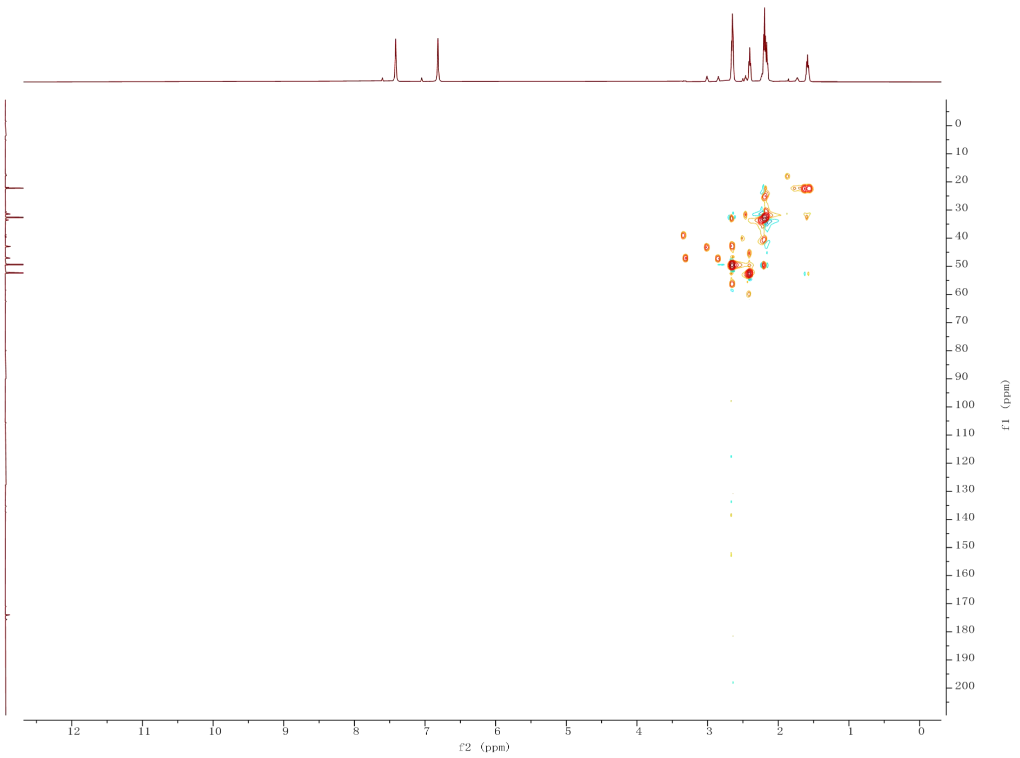

Figure S30　HMBC spectrum of GABA-AA 2 in DMSO-d$_6$.

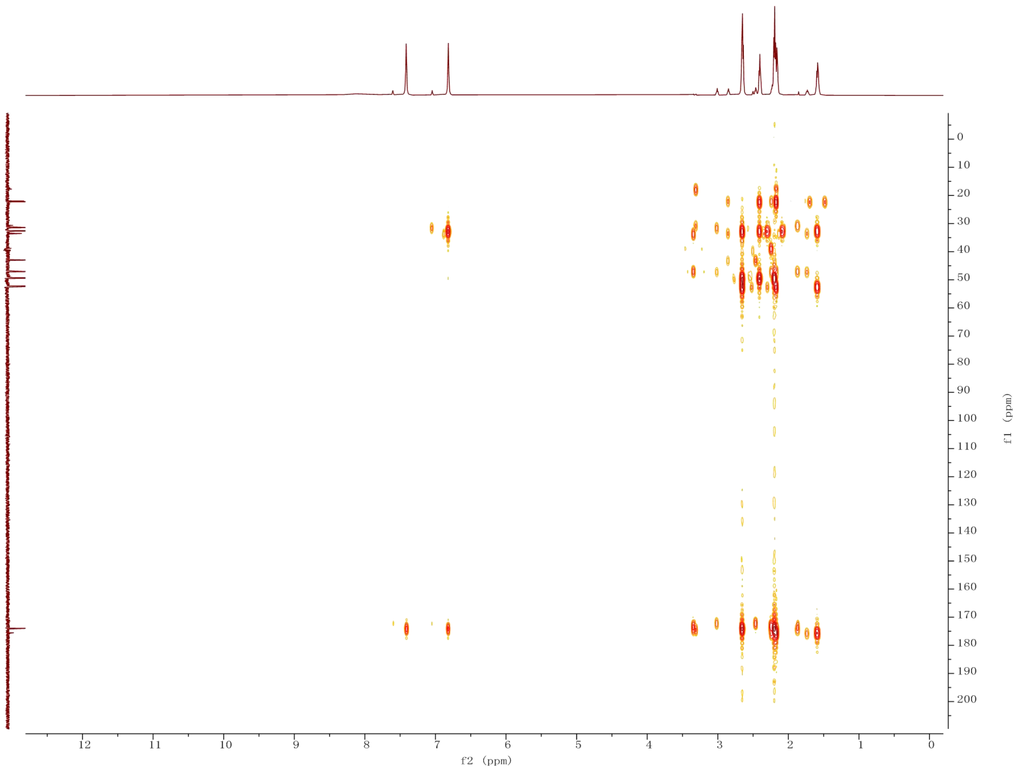

Figure S31 HRMS spectrum of Gly-AA 1.

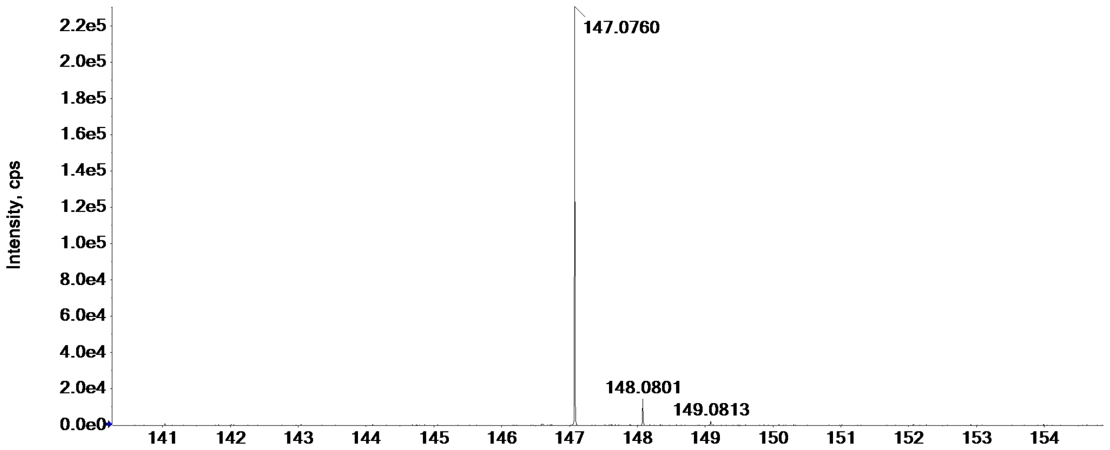

Formula	m/z	RDB	ppm
$C_5H_{10}N_2O_3$	147.0764	2.0	-2.8

Figure S32 ^{1}H NMR spectrum of Gly-AA 1 in D_2O.

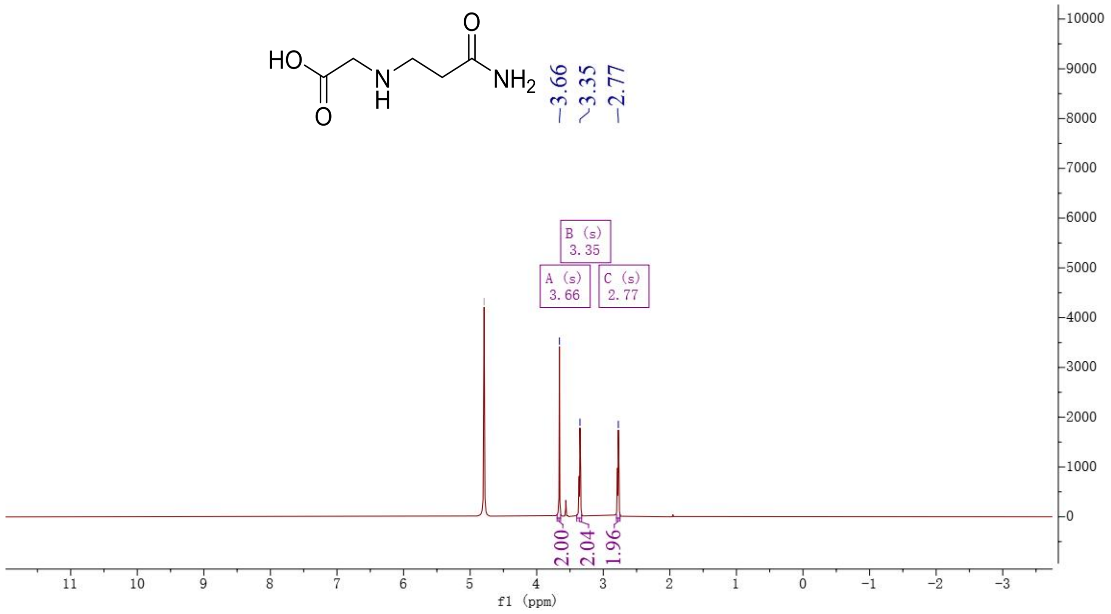

Figure S33 ^{13}C NMR spectrum of Gly-AA 1 in D_2O.

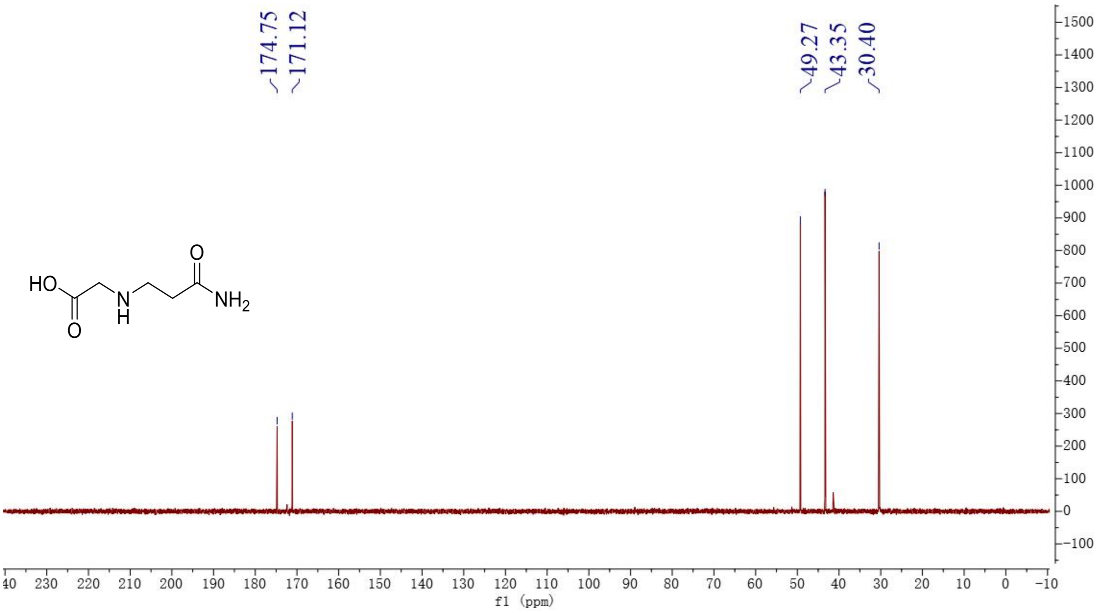

Figure S34 Dept 135 spectrum of Gly-AA 1 in D_2O.

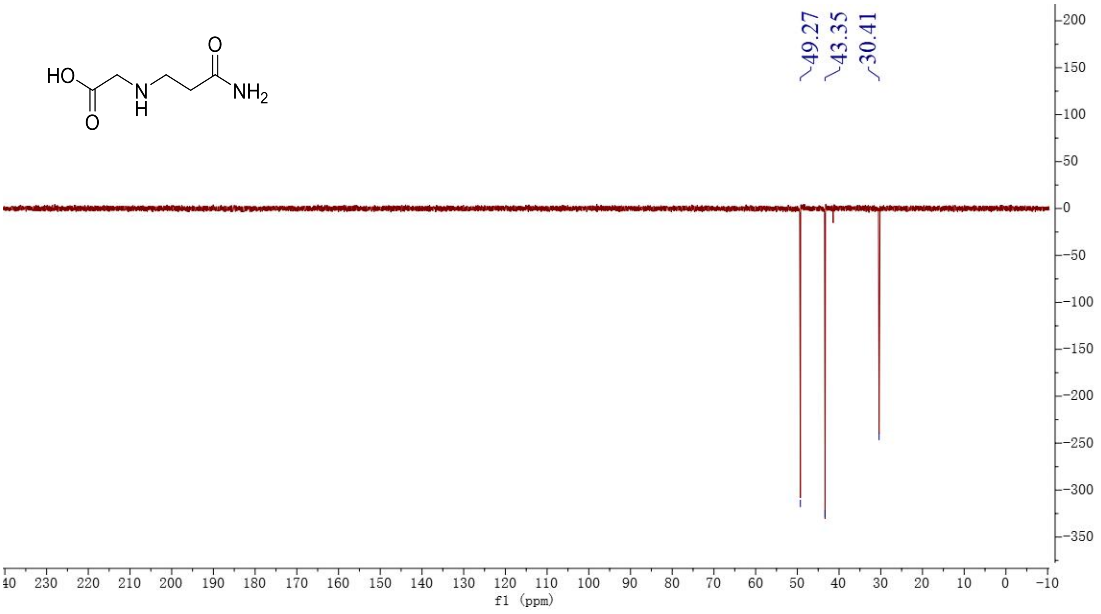

Figure S35 HRMS spectrum of Gly-AA 2.

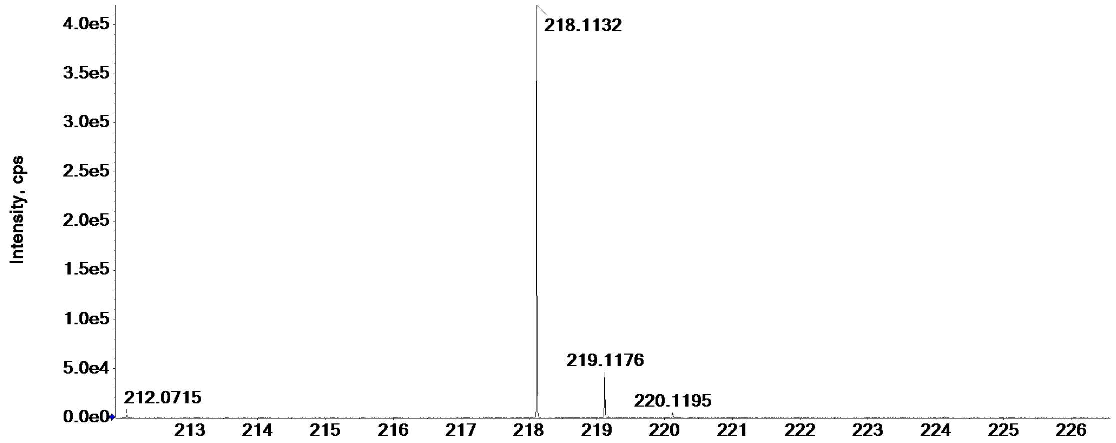

Formula	m/z	RDB	ppm
$C_8H_{15}N_3O_4$	218.1135	3.0	-1.5

Figure S36 ^{1}H NMR spectrum of Gly-AA 2 in D_2O.

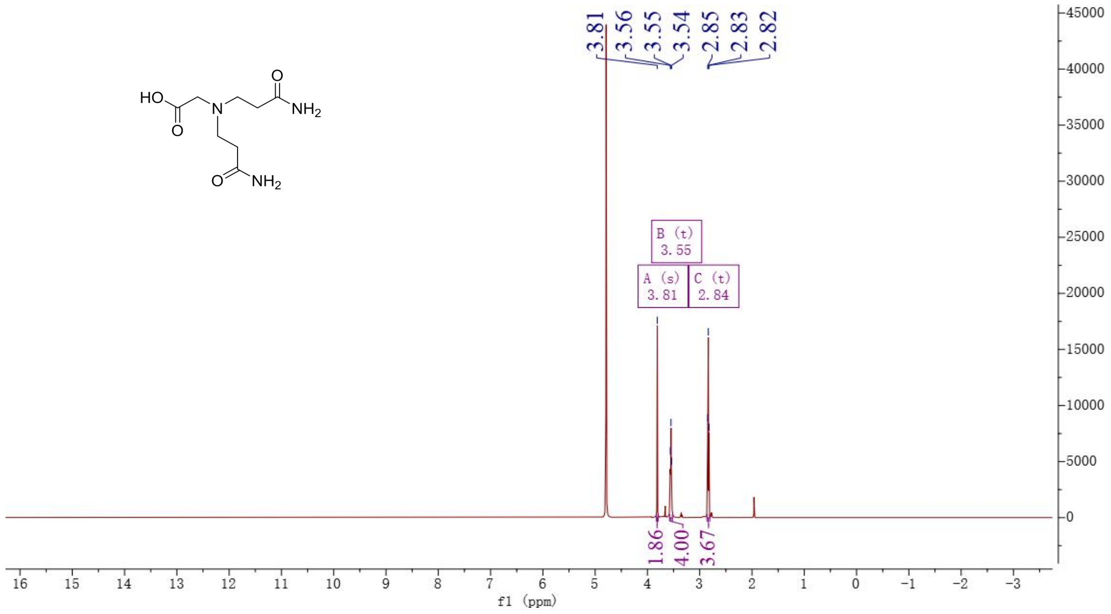

Figure S37 ${}^{13}C$ NMR spectrum of Gly-AA 2 in D_2O.

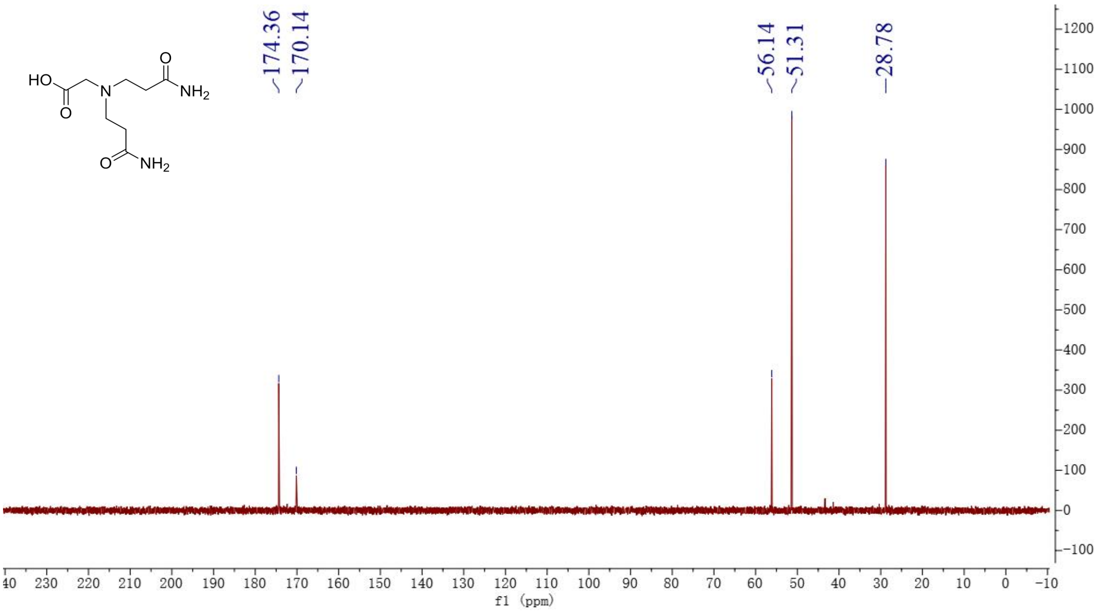

Figure S38 Dept 135 spectrum of Gly-AA 2 in D_2O.

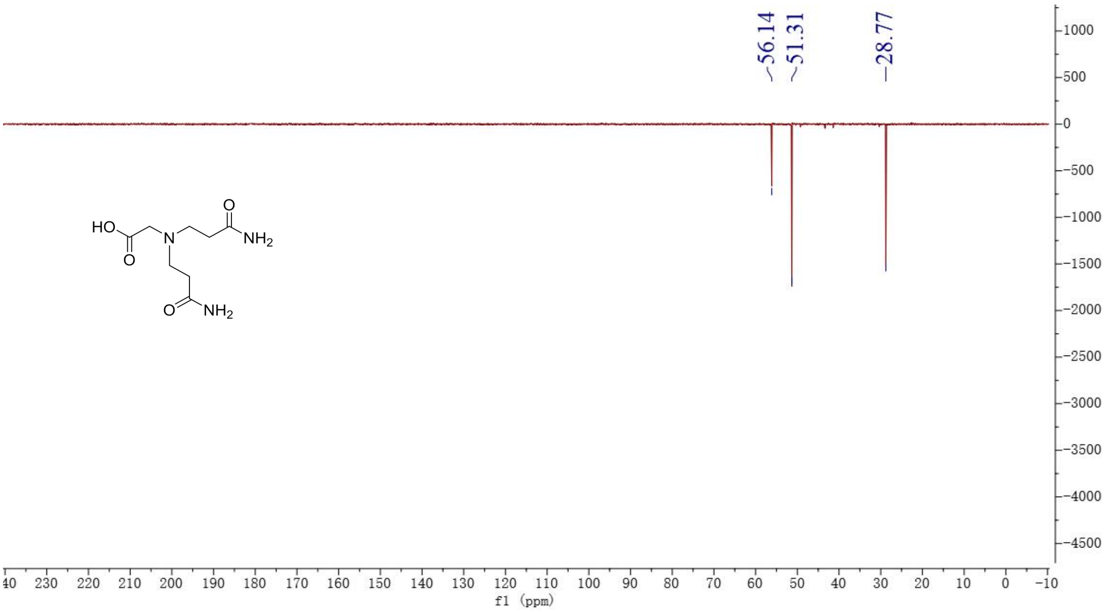

Supplement: Supplementary file 1 [file Data_Sheet_1.PDF]
